# Lipid-Nucleic Acid Complexes: Physicochemical Aspects and Prospects for Cancer Treatment

**DOI:** 10.3390/molecules25215006

**Published:** 2020-10-28

**Authors:** Ricardo Gaspar, Filipe Coelho, Bruno F. B. Silva

**Affiliations:** INL—International Iberian Nanotechnology Laboratory, Av. Mestre José Veiga, 4715-310 Braga, Portugal; ricardo.gaspar@inl.int (R.G.); filipe.coelho@inl.int (F.C.)

**Keywords:** lipid-DNA, lipoplexes, gene therapy, siRNA, mRNA, liposomes, cancer, non-viral

## Abstract

Cancer is an extremely complex disease, typically caused by mutations in cancer-critical genes. By delivering therapeutic nucleic acids (NAs) to patients, gene therapy offers the possibility to supplement, repair or silence such faulty genes or to stimulate their immune system to fight the disease. While the challenges of gene therapy for cancer are significant, the latter approach (a type of immunotherapy) starts showing promising results in early-stage clinical trials. One important advantage of NA-based cancer therapies over synthetic drugs and protein treatments is the prospect of a more universal approach to designing therapies. Designing NAs with different sequences, for different targets, can be achieved by using the same technologies. This versatility and scalability of NA drug design and production on demand open the way for more efficient, affordable and personalized cancer treatments in the future. However, the delivery of exogenous therapeutic NAs into the patients’ targeted cells is also challenging. Membrane-type lipids exhibiting permanent or transient cationic character have been shown to associate with NAs (anionic), forming nanosized lipid-NA complexes. These complexes form a wide variety of nanostructures, depending on the global formulation composition and properties of the lipids and NAs. Importantly, these different lipid-NA nanostructures interact with cells via different mechanisms and their therapeutic potential can be optimized to promising levels in vitro. The complexes are also highly customizable in terms of surface charge and functionalization to allow a wide range of targeting and smart-release properties. Most importantly, these synthetic particles offer possibilities for scaling-up and affordability for the population at large. Hence, the versatility and scalability of these particles seem ideal to accommodate the versatility that NA therapies offer. While in vivo efficiency of lipid-NA complexes is still poor in most cases, the advances achieved in the last three decades are significant and very recently a lipid-based gene therapy medicine was approved for the first time (for treatment of hereditary transthyretin amyloidosis). Although the path to achieve efficient NA-delivery in cancer therapy is still long and tenuous, these advances set a new hope for more treatments in the future. In this review, we attempt to cover the most important biophysical and physicochemical aspects of non-viral lipid-based gene therapy formulations, with a perspective on future cancer treatments in mind.

## 1. Introduction

Cancer usually results from mutations in cancer-critical genes. The wide diversity of genes, cell-types and tissues, their underlying interactions, and secondary mutations caused by the abnormal high-rate cell multiplication, make cancer a very complex disease to understand and treat [1]. The World Health Organization expects 30 million incident cancer cases worldwide in 2040, compared with the already expressive 18 million in 2018, and the mortality rate is expected to be approximately 50% [2]. Hence, novel diagnosing, monitoring and therapeutic methodologies are required to improve the life expectancy and quality of life of cancer patients. Gene therapy approaches are especially attractive because while using similar nucleic acid (NA) technologies, they have the potential of targeting different cancer-critical genes at their root [3,4,5,6,7] or stimulating the patients’ immune system against the disease using immunotherapy approaches [8,9,10,11,12,13,14,15]. This makes gene therapy a versatile and powerful approach. Common NA therapeutic platforms include plasmid DNA (pDNA), messenger RNA (mRNA), small-interference RNA (siRNA), microRNA (miRNA) and antisense oligonucleotides (ODN). Cancer gene therapy strategies can take diverse forms, such as: (i) supplementing tumor suppression genes (e.g., *p53* and *mda-7*/IL-14) with DNA or mRNA delivery [5,7]; (ii) silencing oncogenes with RNA interference (e.g., using siRNA) [6]; and (iii) stimulating the patients’ immune system against the disease with mRNA cancer vaccines encoding tumor antigens [8,9,10,13] or intratumoral vaccines with genes encoding immunomodulatory proteins [16]. The prospect of using the immune system to attack cancer is especially promising since induced effector memory T cells may have the capacity to search for and prevent metastasis. In fact, early-stage clinical trials are starting to show promising results [9,10,11,12,13].

One of the biggest difficulties of gene therapy is the delivery of the therapeutic NAs to their target cells in patients. NAs are highly charged anionic macromolecules that are rapidly cleared from circulation. Hence, they require a delivery vehicle that takes them to the desired location. Depending on the exact therapeutic application, a vehicle-NA system needs to overcome several biological barriers [4,17]. If administered intravenously, to take advantage of eventual enhanced permeation and retention (EPR) effects [18] and meet other clinical requirements [19], particles should have sizes of about 100 nm or less. A typical pDNA with ca. 5 kbp has a length of ca. 1700 nm, needing to be substantially condensed to achieve such size requirements. If administrated i.v. the vehicle-NA assembly also needs to avoid clearance from the blood and reach the targeted organs and tissues. It subsequently has to be uptaken by the target cells, which is usually achieved by endocytosis, and escape from the endosomes [20]. If the therapeutic NA is siRNA or mRNA, these NAs need to be released from the complex into the cell cytoplasm. If the therapeutic NA is DNA, it needs to reach the nucleus to be expressed. This seemingly daunting task is performed extremely well by viruses. This high efficiency achieved by viral capsids remains when the viral NAs are removed and replaced by therapeutic NAs. Hence, viral gene therapy approaches are the most commonly used in the current clinical trials and approved medicines [7,21]. However, despite their efficiency, viral vectors have also several drawbacks, including immunogenicity, limited capacity for loading large genes and poor scalability, which leads to high treatment costs [3,4].

Treatment of common diseases (such as cancer) requires the development of more versatile and affordable non-viral vectors in order to be available to the population at large. Membrane-type lipids exhibiting permanent or transient cationic character are able to associate with NAs via electrostatic interactions, forming nanosized complexes that efficiently transfect eukaryotic cells [22,23]. Such lipid-based systems are especially attractive due to their versatility, low immunogenicity, capacity for loading full-length genes and regulatory sequences (unlike the viral capsid limited capacity), and potential for simpler, more scalable and affordable production [3,24,25,26]. While in many cases their efficiency in vivo is still relatively low, there are also early-stage clinical trials showing promising results [8,15,27]. In fact, the first non-viral gene therapy medicine was recently approved by the US Food and Drug Administration (FDA) for the treatment of hereditary transthyretin amyloidosis, setting the stage for further progress in the future [19]. As advances in lipid-based NA assembly methods and knowledge about the critical in vivo interactions continue accumulating it is our hope that new, more efficient and more general gene therapy formulations for cancer treatment can be discovered. In this review, we attempt to cover the most important physicochemical aspects of non-viral lipid-based gene therapy formulations, with a perspective on future cancer treatments in mind.

## 2. Nucleic Acid Biophysical Properties and Therapeutic Uses

The main therapeutic NAs used in cancer therapy are DNA, siRNA, mRNA and miRNA. Besides the different therapeutic strategies that each of these molecules allow, they also have differences between themselves, which as discussed in Section 4, may influence the properties of the formulation. In this section, we describe the most important physical-chemical aspects of these NAs and give a very brief outline of some of the therapeutic approaches using them.

**Figure 1:**
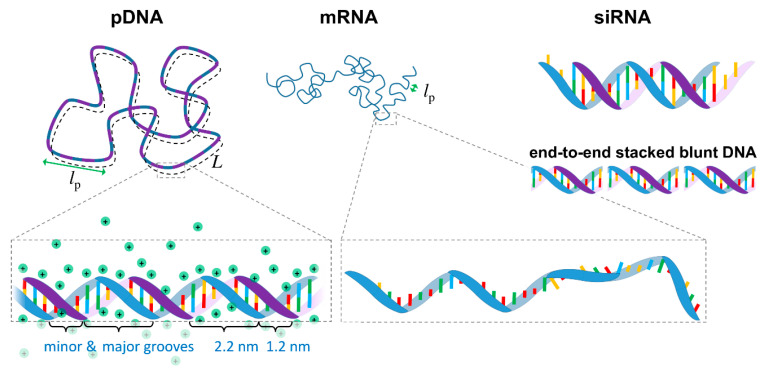
Schematic representation of plasmid DNA (pDNA), messenger RNA (mRNA), small interfering RNA (siRNA) and end-to-end stacked blunt short DNA. The contour length *L* represents the total length of the macromolecules, and the persistence length *l*_p_ represents the length in which the polymer chain remains approximately straight. The *l*_p_ for DNA (double stranded) at moderate ionic strength is ca. 50 nm, whereas for mRNA (single stranded) is ~2 nm. siRNA is an essentially rigid molecule (*l*_p_ > *L*) and typically contains two unpaired bases on each strand. All these polymers are anionic and highly charged. The counterion cloud with a condensed counterion layer closer to the nucleic acid backbone (Manning condensation) is explicitly shown for pDNA and omitted for the remaining nucleic acids (NAs) for clarity. Small DNA fragments without unpaired bases (blunt) stacked end-to-end via hydrophobic interactions between the nucleobases are also shown.

From a biophysics perspective, NAs can be seen as highly charged anionic polymers (anionic polyelectrolytes). Most polymers can, to some extent, be described by a simplified worm-like chain model, in which the polymer chain is described by a contour length *L*, and a persistence length *l*_p_, which is a measure of the polymer rigidity (i.e., the length in which the polymer remains roughly straight, Figure 1).

### 2.1. DNA

DNA is typically found in a double stranded configuration (dsDNA) in which two single stranded DNA (ssDNA) chains with complementary base sequences are joined together by hydrogen bonds and hydrophobic interactions between the bases, forming a double helix conformation [28]. It is found most commonly in the B-form, in which the two helices are arranged in a minor and major grooves, with the sugar-phosphate backbones in each being distanced by ca. 1.2 and 2.2 nm, respectively (Figure 1). dsDNA can be described as a semiflexible polymer. Its diameter is 2.07 nm, and the distance between the base pairs is 0.34 nm [28], resulting in a high linear charge density of -2*e*/0.34 nm. The *l*_p_ is ca. 50 nm at moderate ionic strengths [29,30,31]. As such, DNA is a relatively rigid and highly charged macromolecule. Because of the high charge density, ca. 76% of the DNA sodium counterions are expected to be located very closely to the phosphate groups to neutralize part of the charge, in what is referred to as Manning condensation [30,31,32,33] (Figure 1). Due to the interactions between bases, DNA can also undergo end-to-end stacking into longer chains. This is especially visible in short dsDNA with complementing unpaired base overhangs or blunt ends (Figure 1), which form longer chains held by intermolecular interactions strong enough to induce the formation of DNA liquid crystals in 2D and 3D [34,35].

Therapeutic DNA is typically used in the form of plasmids (pDNA) encoding whole genes and regulatory sequences for endogenous proteins, such as the suppressor gene *p53* [36]. This is the most frequently mutated gene in human cancer and consequently the most commonly transferred suppressor gene in clinical trials [7]. In some cases, the plasmids encode exogenous proteins such as oncolytic viral proteins to induce tumor cell apoptosis and inhibit tumor growth [37]; exogenous enzymes (also termed “suicide genes”) that convert non-toxic pro-drugs into cytotoxic drugs selectively within the tumor [38,39]; or cancer antigens to stimulate the immune system in cancer immunotherapy [40]. All these strategies are being tested in clinical trials [7]. DNA can also be used to express DNA endonucleases such as Cas9 to edit genes through the CRISPR mechanism [41], in which case the plasmids contain the information to express the Cas9 protein and to encode the RNA guide strand with the targeted gene sequence [42].

### 2.2. siRNA

siRNA is a double stranded RNA with a relatively short length of typically 19–25 bp (plus two non-paired nucleotide overhangs at each 3′ side—Figure 1). Because of the different sugar backbone, siRNA is in the A-conformation, which means that the double helix diameter is slightly larger than DNA (ca. 2.6 nm [28,43]), and the distance between base pairs is smaller (ca. 0.27–0.29 nm [43,44]), resulting in a higher linear charge density of ca. -2*e*/0.29 nm than DNA. The *l*_p_ for dsRNA is 63–72 nm [43,44] for moderate ionic strength, reflective of the higher rigidity compared to DNA. Given the small siRNA size compared with its *l*_p_, this means that siRNA behaves essentially as a stiff short rod. The helix conformation and its associated counterion distribution seem to be more effective at screening the charge on siRNA than on DNA [45].

Therapeutic siRNA is used to silence genes using the RNA interference pathway [46]. Very briefly, the siRNA activates the RNA-Induced Silencing Complex (RISC), which takes the guide strand (anti-sense) of siRNA as a template to cut in a catalytic way mRNA with a complementary base sequence to the guide strand. Hence, the protein encoded by the mRNA is not expressed and the gene is silenced. This approach is especially attractive for the treatment of autosomal dominant disorders, such as is the case of the first lipid-based gene therapy medicine approved for clinical use [19]. In the context of cancer, the potential to silence the expression of any protein involved in tumor initiation and progression is also highly sought [47], for instance targeting specific oncogenes [48], or proteins overexpressed in tumors [49]. One particularly interesting aspect of siRNA delivery in the cancer context is the possibility to deliver multiple siRNA molecules to target multiple genes involved in cancer. This takes advantage of the fact that the physical properties of different siRNAs are similar and amenable for co-encapsulation [6]. This approach was demonstrated already in a phase I clinical trial using lipid nanoparticles containing siRNA targeting the VEGFA and KSP genes, both of which are overexpressed in a variety of tumors [50]. Targeting VEGFA is proposed to reduce tumor angiogenesis, while KSP is essential for cell division in proliferative cells. A few early-stage clinical trials have been concluded showing overall favorable results, namely, the safety of the formulations and general ability of lipid-based particles to deliver siRNA to tumors [6,50,51,52,53].

### 2.3. mRNA

In contrast with the therapeutic DNA and siRNA, mRNA is a single stranded oligonucleotide, typically containing several hundreds of nucleotides (Figure 1). Because it is single stranded, mRNA is much more flexible than DNA. The average distance between phosphates is ca. 0.62 nm, but neighboring purine bases (A and G) can still stack, resulting in sections that are more rigid with an average distance between bases of ca. 0.37 nm [54]. Overall, the *l*_p_ for physiologic ionic strengths is below 2 nm [54,55,56], being much more flexible than the double stranded NAs. mRNA is also slightly more flexible than single stranded DNA [55,56]. While the linear charge density decreases by a factor slightly greater than two compared to dsDNA, mRNA is still a highly charged macromolecule. Importantly, because the bases are now more exposed, in analogy with single stranded DNA [57,58,59,60], it is expectable that mRNA also has a stronger amphiphilic character than dsDNA, which can lead to additional hydrophobic interactions with the lipids beyond the expected electrostatics.

One of the greatest advantages of mRNA over DNA for gene therapy is that mRNA is expressed in the cell cytoplasm, thus avoiding the difficulty of the NA translocation to the cell nucleus. mRNA also does not integrate into the genome, posing no risk of mutagenesis and is readily degraded in serum, reducing safety concerns [61]. mRNA is, however, in its native form extremely sensitive to nucleases and more immunogenic than DNA. Fortunately, some chemical modifications on the RNA backbone can improve both of these aspects [12,62]. As with therapeutic DNA, mRNA can be used to express endogenous proteins that may be missing in the patient due to faulty genes, or to express exogenous proteins such as Cas9 for gene editing. In the latter, along with the mRNA encoding Cas9, the guiding mRNA strand that directs the editing also has to be delivered [63]. Currently, one of the most exciting and promising uses of mRNA gene delivery is in immunotherapy [11,12]. These include mRNA cancer vaccines encoding cancer antigens [8,64] and intratumoral delivery of mRNA encoding immunomodulatory proteins [16] to stimulate the patients’ own immune system to fight cancer. One particular highlight, made possible by advances in sequencing and mRNA synthesis, is the possibility to quickly and safely produce personalized mRNA cancer vaccines encoding cancer neoantigens specific for each patient [9,10,12,13]. The identification of each patient’s mutations most likely to activate T cells against the tumor has led to promising results in early-stage clinical studies [9].

### 2.4. miRNA

microRNAs (miRNAs) are small (~23 nucleotides), single-stranded, non-coding RNAs derived from ~70 nucleotide hairpin-forming miRNA precursors (pre-miRNAs) [65,66]. Malfunctions in miRNAs are associated with tumor formation and progression through regulation of the expression of critical genes. Such genes can be involved in processes such as cell cycle, metabolism, apoptosis, angiogenesis, metastasis and immunosuppression. Hence, depending on the specific tumor type, miRNAs can be seen as therapeutic agents or therapeutic targets. In the former, the therapeutic strategy consists of supplementing functional miRNA mimics, whereas in the latter, the strategy consists of administering miRNA antagonists [66,67]. One particularly appealing aspect of miRNA to fight cancer, which typically involves several genes, is that miRNAs not only can silence target genes efficiently, but also simultaneously regulate a broad range of genes of interest via imperfect base-pairing of multiple sequences [66]. Lipid-based nanoparticles loaded with both miRNA mimics and antagonists have shown promising pre-clinical results [68,69,70]. Regarding clinical trials it is still too early to draw significant conclusions. The first early-stage clinical study was halted due to serious immune responses [71], while the second provided overall positive results in terms of safety and early signs of activity [72], providing optimism on further developments in the field.

## 3. Lipid Self-Assembly and Lipid Film Elastic Energy

**Figure 2:**
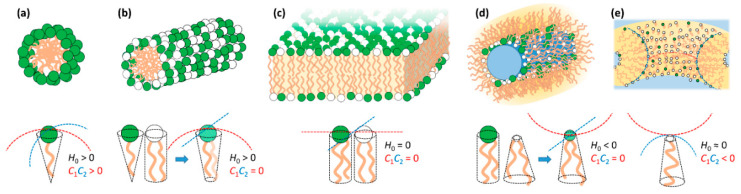
Schematic representation of typical self-assembly topologies in mixtures of cationic (green) and neutral/zwitterionic (white) lipids. In the bottom the respective normal spontaneous (*H*_0_) and Gaussian (*C*_1_*C*_2_) curvatures along with their associated lipid molecular shapes are shown. When no oppositely charged lipids are present, as is the case, the lipid mixture curvatures result from the combination of the curvatures of the individual lipids. (**a**) Spherical micelle, typical of surfactant solution and normal cubic phases. Favored by surfactants with large positive membrane curvature. (**b**) Elongated micelle, typical of surfactant solution and normal hexagonal phases. Favored by surfactants with positive curvature. (**c**) Bilayer, characteristic unit structure of liposomes and lamellar phases. Favored by lipids with zero spontaneous curvature. (**d**) Reverse elongated micelle, typical of inverted hexagonal phases. Favored by lipids with negative curvature such as DOPE. (**e**) Bilayer in a saddle-type arrangement, typical of sponge phases, bicontinuous cubic phases and pore structures. Favored by lipids with normal zero but negative Gaussian curvatures.

Surfactants and membrane-type polar lipids, which here we refer to simply (and collectively) as lipids, are amphiphilic molecules that tend to self-assemble in water to avoid contact of the hydrophobic tails with the solvent [73]. The structures of the assembled phases can be understood by the spontaneous curvature (*H*_0_) of the lipid mixture. If the lipid film prefers to curve towards the hydrophobic tails (*H*_0_ > 0), it tends to form normal phases such as normal spherical and elongated micelles; if it curves towards the headgroup area and water (*H*_0_ < 0), it tends to form reverse phases such as inverted micelles; if it remains flat (*H*_0_ = 0), it tends to form bilayers (Figure 2). The spontaneous curvature is intrinsically related with the molecular shape of the lipids, that is, whether they resemble a normal cone with the headgroup at the base (*H*_0_ > 0), a cylinder (*H*_0_ = 0) or a truncated cone with tail at the larger base (*H*_0_ < 0).

### 3.1. Lipid Film Elastic Energy

A powerful description of lipid phase behavior can be obtained by the local curvature free energy *g*_C_, which is described, to first order, by [74]:

(1) $$g_{c}=2\kappa {\left(H-H_{0}\right)}^{2}+\kappa_{G}C_{1}C_{2}$$

Here, *H* = (*C*_1_ + *C*_2_)/2, is the normal curvature and *C*_1_ = 1/*R*_1_ and *C*_2_ = 1/*R*_2_ are the two local principal curvatures. *H* = *H*_0_ at equilibrium. κ and κ_G_ are the bending and saddle splay moduli, respectively, and *C*_1_*C*_2_ is the Gaussian curvature. The value of κ indicates how energetically costly it is to bend the lipid membrane away from *H*_0_ (i.e., how rigid is the membrane). For reference, a typical bilayer, composed of POPC, has a κ of ca. 25 *k*_B_*T* at moderate salt concentration and room temperature [75]. Here, *k*_B_*T* is the thermal energy, where *k*_B_ is the Boltzmann constant and *T* the absolute temperature.

Importantly also, when κ_G_ > 0, the formation of structures with negative Gaussian curvature (e.g., bicontinuous cubic phase and channels/pores connecting two membranes) becomes energetically favored. As will be seen below, this is of relevance for the escape of lipid-NA particles trapped inside endosomes (Section 6) and for the formation of lipid-siRNA cubic phases (Section 4). Monoolein (MO) and phytantriol are two common lipids with κ_G_ > 0.

### 3.2. Lipid Mixtures

**Figure 3:**
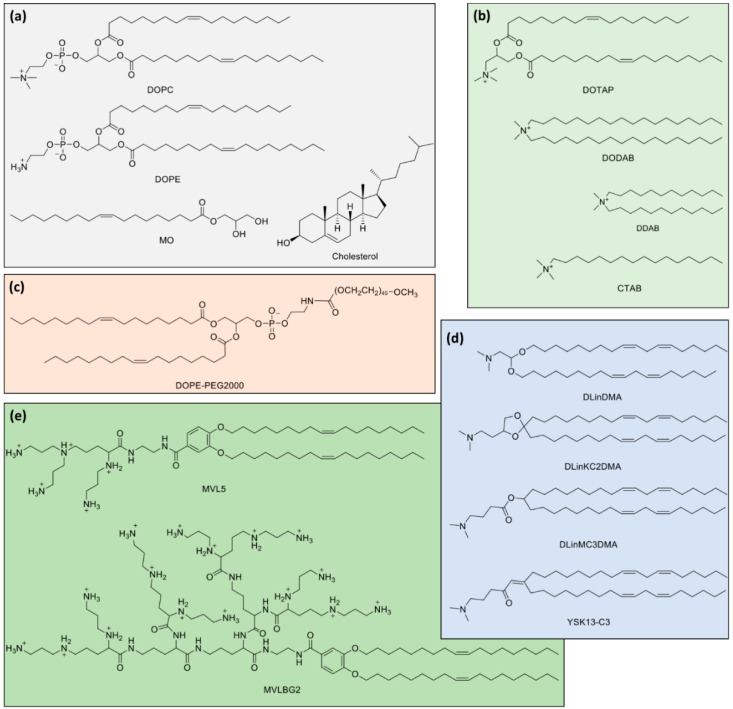
Molecular structures of lipids used in gene therapy formulations and discussed throughout the text. (**a**) Typical zwitterionic (1,2-dioleoyl-*sn*-glycero-3-phosphocholine (DOPC) and 1,2-dioleoyl-*sn*-glycero-3-phosphoethanolamine (DOPE)) and neutral lipids (monoolein (MO) and Cholesterol). (**b**) Typical cationic lipids (1,2-dioleoyl-3-trimethylammonium propane (DOTAP), dimethyldioctadecylammonium bromide (DODAB), didodecyldimethylammonium bromide (DDAB) and cetyltrimethylammonium bromide (CTAB)). (**c**) PEGylated lipid with DOPE backbone and PEG M_w_ of ca. 2000 Da. (**d**) Ionizable cationic lipids (1,2-dilinoleyloxy-3-dimethylaminopropane (DLinDMA), 2,2-dilinoleyl-4-(2-dimethylaminoethyl)-[1,3]-dioxolane (DLinKC2DMA), O-(*Z,Z,Z,Z*-heptatriaconta-6,9,26,29-tetraen-19-yl)-4-(*N,N*-dimethylamino)butanoate (DLinMC3DMA) and 2-(dimethylamino)propyl-(12*Z*,15*Z*)-3-((9*Z*,12*Z*)-octadeca-9,12-dien-1-yl)henicosa-2,12,15-trienoate (YSK13-C3). (**e**) Multivalent cationic lipid *N*1-[2-((1*S*)-1-[(3-aminopropyl)amino]-4-[di(3-amino-propyl)amino]butylcarboxamido)ethyl]-3,4-di[oleyloxy]-benzamide (MVL5, nominal charge of +5) and dendritic headgroup cationic lipid *N*1-2-[((1*S*)-1,4-di[(1*S*)-1,4-di((1*S*)-1,4-di[(3-aminopropyl)amino]-butylcarboxamido)butyl]carboxamidobutyl)carboxamido]ethyl-3,4-di[(*Z*)-9-octadecenyloxy]benzamide (MVLBG2, nominal charge of +16).

Most lipid systems used in lipid-NA particles consist of at least two lipids: one cationic and one neutral/zwitterionic lipid. Some examples of commonly used lipids can be seen in Figure 3. Whereas one of the important functions of cationic lipids is to complex/condense the NAs, the neutral (also called “helper”) lipid allows modulating the normal spontaneous and Gaussian curvatures, as well as the bending rigidity κ. Importantly, the molar fraction of neutral lipid also modulates the membrane charge density σ_M_, which as will be seen below, influences the degree of DNA packing and interactions with the endosomal membrane for activated fusion. In many cases, the *H*_0_ of a mixture can be reasonably approximated by the mean of the *H*_0_ of the individual lipids (e.g., addition of a bilayer forming lipid to a globular micelle forming lipid may result in elongated micelle formation—Figure 2b). In some other cases, e.g., when having oppositely charged lipids, deviations are striking due to the synergistic electrostatic interaction between the two headgroups [76,77].

The lipid membrane charge density σ_M_ is directly proportional to the fraction of cationic lipid in the lipid bilayer and to the nominal charge (*Z*) per lipid according to:

(2) $$\sigma_{M}=ZeN_{cl}/\left(N_{cl}A_{cl}+N_{nl}A_{nl}\right)$$

where *e* is the elementary charge, *N*_*cl*_ and *N*_*nl*_ are the number of cationic and neutral lipids, respectively, and *A*_*cl*_ and *A*_*nl*_ are their respective molecular areas in the bilayer. The σ_M_, while often overlooked, is a crucial parameter in the understanding of lipid-NA systems and their interactions with cells, as will be described in Section 6.

## 4. Lipid-NA Nanoparticle Formation, Structure and Stability

Lipid-NA nanoparticles (or lipoplexes) readily form for a variety of lipids and nucleic acids [22,23]. This constitutes one of their main advantages: ease of formation and versatility [78]. In this section we attempt to provide a simplified fundamental description about their formation, structure and stability.

### 4.1. Lipid-NA Complexation: Counterion Release as the Main Driving Force for Lipid-NA Association

**Figure 4:**
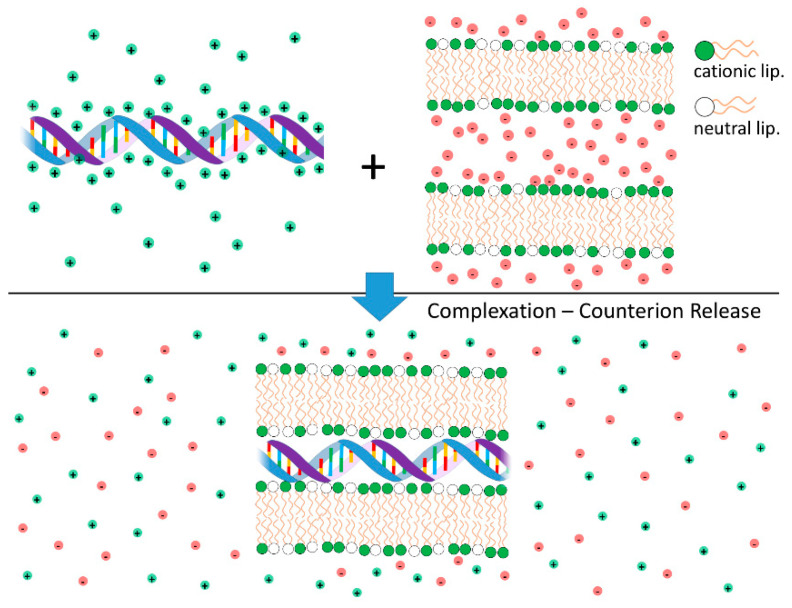
Schematic illustration of the release of inorganic counterions associated with cationic liposome-DNA complexation and consequent increase of the system entropy. On the top are shown segments of non-complexed DNA (anionic) and cationic bilayers. Shown also are their inorganic counterions, with limited mobility and in close proximity to the charged surfaces. On the bottom, a segment of an isoelectric complex is shown. As the oppositely charged DNA and cationic bilayers neutralize each other, the inorganic counterions are released to the surrounding medium, leading to a high increase in the system entropy. This entropic gain is the main contribution to the complexation free energy.

Cationic liposomes (CLs) and DNA interact strongly leading to the formation of complexes with well-organized structures. The electrostatic free energy driving complexation between the two species has two main contributions: (i) Coulomb attractions between cationic liposomes and nucleic acids (anionic); and (ii) the large gain in entropy that results from the release of the inorganic counterions from both cationic lipid and DNA when these two species partially neutralize each other [79,80,81] (Figure 4). Indeed, this second component is the major driving force for complexation. These findings have been convincingly demonstrated through calorimetric measurements, where the enthalpy change involved in the complexation between CLs and DNA was found to be endothermic (thus requiring an increase in entropy to drive complex formation) [82], and by measurements of conductivity to count the number of released ions [83]. Even when used qualitatively, the concept of counterion release provides a strong insight into the overall lipoplex phase behavior. For instance, this framework helps explaining why anionic liposomes and DNA form complexes in the presence of divalent inorganic salt (e.g., Ca^2+^) [84]. Since a divalent cation can simultaneously neutralize one negative charge in both the DNA and anionic liposome, two of the original monovalent counterions can be released, leading to a net gain in entropy and complexation. A similar mechanism can also explain the formation of complexes between zwitterionic liposomes and DNA in the presence of Ca^2+^ [85,86]. The formation of complexes driven by counterion release is a widespread phenomenon in colloid science [76] and in biology [31,81,87].

### 4.2. Cationic-to-Anionic Charge Ratio, Overcharging and Colloidal Stability of Lipid-NA Complexes

The cationic-to-anionic charge ratio, here represented as ρ_chg_, is a critical parameter influencing the structure, lipoplex colloidal stability and their interactions with biologic systems [3,25]. ρ_chg_ is defined by the total number of cationic charges coming from the lipid system divided by the total number of anionic charges coming from the NA. An analogous term, the N/P ratio, is also commonly used, referring to the ratio of amine groups from the cationic lipid to the number of phosphate groups from the NA [26].

The counterion release in lipoplex formation is maximum, and almost complete when ρ_chg_ ~1, around the particle isoelectric point. Such particles have a near net-zero charge, typically showing poor colloidal stability and formation of large aggregates [79]. For ρ_chg_ greater or smaller than one, the lipoplexes become overcharged with a net positive or negative charge, respectively [88]. The extent of lipoplex overcharging depends on the lipoplex structure and lipid membrane charge density (σ_M_) as discussed further below. Typically, lipoplexes can become more positively overcharged if lipid membranes have higher σ_M_. Conversely, lipid membranes with lower σ_M_ can become more negatively overcharged [88]. It is also plausible to expect differences in overcharging degree between DNA, siRNA and mRNA, as observed for different polyelectrolytes [89,90,91]. Further addition of liposomes or DNA beyond the maximum overcharge leads to the coexistence of lipoplexes with either liposomes or DNA in excess. The overcharging behavior can also be rationalized within the counterion release framework. In general, it can be stated that additional incorporation of a liposome or DNA into a complex leads to overcharging as long as the charge density of the resulting overcharged complex is smaller than the original liposome or DNA before binding [92]. This means that the counterions loosely bound to the resulting overcharged complex have more mobility (hence more entropy) than the counterions of the liposome or DNA before complexation [79].

Importantly, the ρ_chg_ is a critical parameter regarding the interactions of lipoplexes with biological systems [8,26,78,93] as will be described in better detail in Section 6.

### 4.3. Lipid-NA Structure

DNA is a rigid and long molecule. mRNA is typically somewhat shorter than DNA plasmids and significantly more flexible due to being a single strand. Further, because of the single-stranded nature, the exposed nucleobases make mRNA significantly hydrophobic. siRNA is a rigid but short molecule (Figure 1). All these nucleic acids are highly charged. Lipids can have positive, neutral or negative spontaneous curvature (*H*_0_), with typical bending rigidities κ significantly larger than the thermal energy *k*_B_*T*. This means that lipids tend to organize in structures that favor their *H*_0_, such as micelles, bilayers and inverted micelles, as discussed in Section 3 (Figure 2).

When cationic liposomes and NAs come into contact, they maximize the gain of entropy from counterion release, while accommodating elastic requirements from both the lipid film and NAs (cf. Section 2 and Section 3). Given the diverse nature of lipidic systems and NAs, several structures are found for lipid-NA systems. Importantly, these structures result in different interactions with cells and different transfection efficiencies (cf. Section 6). Knowledge of the main factors leading to these structures and how to tune them, leads to knowledge of how to improve their therapeutic potential.

**Figure 5:**
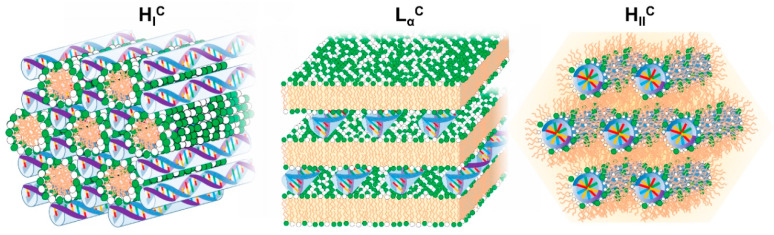
Schematic illustration of the most common structures found for cationic lipid-DNA assemblies. On the left, the normal hexagonal phase (*H*_I_^C^) is shown, with lipid elongated micelles arranged on a hexagonal lattice and the DNA rods arranged on a honeycomb lattice in the interstices between the lipid micelles [97]. In the middle is the lamellar phase (*L*_α_^C^), with alternating lipid bilayers and DNA monolayers sandwiched between them [79]. On the right is the inverted hexagonal phase (*H*_II_^C^) with lipid inverted micelles coating the DNA arranged on a hexagonal lattice [98].

Overall, the electrostatic free energy (which accounts for both Coulomb attraction and counterion release) is maximized if the cationic lipid is able to fully wrap the NA double helix. However, the elastic energy required to bend the lipid membranes and NA chains also has to be taken into account. The resulting lipid-NA structures will represent a compromise between these three most dominant energetic terms (electrostatic interactions and elasticity from both NA and lipid film). Due to the relatively high rigidity of the DNA double helix, structures that favor a suitable contact between the cationic lipids and DNA without bending DNA significantly will be favored. This is the case for the most common multilamellar phase (*L*_α_^C^) and normal and inverted hexagonal phase (*H*_I_^C^ and *H*_II_^C^) structures [94]. Regarding the lipid membranes, if their bending rigidity κ is significantly greater than the thermal energy (*k*_B_*T*), the elastic free energy of the lipid will also be significant and the structure of the complex will be governed by the symmetry of the lipid phase [95] (Figure 5). In other words, the curvature of the lipid membrane will define if the lipoplex will have a *L*_α_^C^, *H*_I_^C^ or *H*_II_^C^ structure. If κ is significantly lower, close to *k*_B_*T*, deforming the lipid membrane is less costly and a *H*_II_^C^ structure is favored, since it optimizes the contact between the cationic lipids and DNA. If siRNA is used instead of DNA, since it is significantly shorter, it allows the formation of bicontinuous cubic phases if that is the preferred arrangement of the lipid phase [96]. Below, we overview most of the structures resolved so far for lipid-NA systems.

#### 4.3.1. Lamellar Complexes—*L*_α_^C^

The *L*_α_^C^ is favored by lipid membranes with spontaneous membrane curvature *H*_0_ = 0. As seen above (Section 3), this zero curvature can be accomplished with many different lipid combinations, including with lipids that want to impose negative curvature such as 1,2-dioleoyl-sn-glycero-3-phosphoethanolamine (DOPE) and MO. The *L*_α_^C^ structure consists of stacked lipid membranes with DNA chains intercalated between them (Figure 5). This structure is able to provide favorable electrostatic interactions between the DNA and cationic membranes while satisfying the elastic requirements of both. That is, the DNA molecule is allowed to remain relatively rigid without excessive bending, and the lipid membranes are allowed to keep their zero mean curvature. The lipid membranes are typically composed of a mixture of cationic and neutral lipids. Even within this flat membrane arrangement, because the lipid chains are typically in the liquid state, cationic lipids tend to undergo local demixing and accumulate preferentially in the vicinity of the DNA rods. This leads to enhanced Coulomb interactions and maximizes entropy from counterion release at the cost of a smaller entropic penalty from the local demixing of the lipid [80,81]. The *L*_α_^C^ structure was convincingly demonstrated by small-angle X-ray scattering (SAXS) measurements [79] and cryogenic transmission electron microscopy (cryo-TEM) [99,100,101]. The X-ray data further reveals that the DNA macromolecules between the lipid bilayers are organized in a 2D smectic phase [79,88,102], in which the average distance between neighboring DNA chains is tunable by the lipid membrane charge density σ_M_ (Equation (2)) and ρ_chg_. This is confirmed by fluorescence cross-correlation spectroscopy (FCCS) measurements, which show that for constant ρ_chg_, higher σ_M_ leads to a larger number of DNA plasmids per particle, while for fixed σ_M_, a higher ρ_chg_ leads to a smaller number of DNA plasmids per particle since there are more cationic particles on where to distribute the DNA [103]. The existence of divalent cations in solution can also lead to further condensation of DNA [104].

In some cases, the positions of the DNA rods are coupled across layers, giving rise to ordered 3D phases [86,100,105,106,107]. Most of these ordered 3D phases have been also identified with SAXS, and were mostly found when the lipid membranes are in the gel phase [86,105,106]. The charge density mismatch of the lipid membranes in the gel phase coupled with reduced lipid mobility may lead to non-optimal shielding of the DNA charges, which leads to increased repulsions between DNA chains across lipid membranes [106]. In the liquid crystalline state, DNA-correlations across layers were only observed so far when short DNA strands (48 bp or lower) are used instead of long DNA chains [107]. These short DNA strands with blunt ends are able to stack end-to-end (Section 2, Figure 1), forming long rods of polyelectrolyte that are effectively longer than the persistence length of long DNA [34,35]. These long lengths of stacked DNA allow the cationic lipid membrane to distort and partially wrap the DNA (to maximize counterion release [81,108]), producing coherent layer-to-layer undulations that result in a 3D columnar phase [107]. What is also interesting is the fact that if the bending rigidity of the cationic membrane κ is lowered by the addition of hexanol (a cosurfactant), the elastic energy cost involved in the deformation of the lipid bilayer is lowered, leading to improved layer-to-layer correlations and a more ordered 3D columnar phase. As pointed out at the beginning of this section, the electrostatic free energy is maximized if the cationic lipid is able to fully wrap the NA double helix, and indeed, if κ is lowered enough, the lipid film can fully wrap DNA and form an inverted hexagonal phase as will be discussed below [98].

The liposome rigidity [109] and the DNA length [110] have been suggested to influence also the overall morphology of lamellar complexes. Lamellar lipoplex formation involving pre-formed liposomes implies the collapse or rupture of the latter to form the alternating DNA-lipid membrane layers. If the liposomes are too rigid, they may resist rupture and just aggregate without forming multilayered lamellar structures [109]. If the liposomes are more flexible, they will rupture or collapse to form lamellar complexes. Here also, the morphologies can vary depending on the system used. In some cases, such as those using liposomes constituted by 1,2-dimyristoyl-sn-glycero-3-phosphocholine (DMPC) and 3*β*-[*N*-(*N′*,*N′*-(dimethylaminoethane)-carbamoyl) cholesterol (DC-Chol), the main morphology consists of multilayered lamellar complexes in which incomplete bilayers are deposited on top of an intact template liposome [111]. In other cases, either by the collapse of the template liposome, or through successive binding and rupture events, the morphology of the complexes is lamellar throughout the particle, from the core to the surface [109,110,112]. Besides the rigidity, other aspects are linked with an influence on the overall morphology, such as DNA length [110], liposome size [113,114], charge ratio ρ_chg_ [109] and formation pathway [109,114,115,116,117]. A comprehensive review on the kinetic aspects and their influence on the structure of lipid-NA complexes can be found in Ref. [109].

The *L*_α_^C^ also forms when CLs are mixed with siRNA [113,118,119]. In contrast with lipid-DNA lipoplexes, the SAXS patterns of siRNA lipoplexes using 1,2-dioleoyl-3-trimethylammonium propane (DOTAP) or multivalent cationic lipid MVL5 (Figure 3e) barely show siRNA-siRNA correlation peaks, which suggests that there is no end-to-end stacking. This is consistent with a 2D liquid-like correlation behavior of the short siRNA rods in contrast with the 2D smectic and 3D columnar arrangements from the long and short DNA systems [118]. Another interesting observation is that, perhaps due to the 2D liquid-like nature of the siRNA organization embedded in the *L*_α_^C^, the overall structure seems to equilibrate more rapidly. This is suggested by the observation of narrower Bragg peaks with SAXS, which also indicates more ordered structures [118].

In contrast to the observation of lack of in-plane order of the siRNA molecules by SAXS, cryo-TEM imaging shows that some lamellar complexes composed of multivalent aminoglycoside-based cationic lipids display fine striations along the lamellae, presumably from ordered siRNA molecules [113]. It is unclear if this may be a specific effect due to the nature of the cationic lipid used. In this family of compounds, changes in the nature of the headgroups while keeping the alkyl backbone constant led to a change in morphology from concentric onion-like structures to more irregular lamellar structures.

Importantly, it was found that compared to DNA, efficient gene silencing by *L*_α_^C^ siRNA lipoplexes required charge ratios (ρ_chg_) nearly an order of magnitude greater, which renders them more toxic. The use of multivalent cationic lipids allows a significant reduction of the number of cationic lipids at a given ρ_chg_, exhibiting lower toxicity and superior silencing efficiency [118]. Cationic surfactants of the gemini type also form lamellar lipoplexes [120].

Some ionizable lipid nanoparticles encapsulating siRNA have been shown to be highly potent in gene silencing [19] and under some conditions seem to show structural features resembling lamellar structures [119,121]. Given their more specific nature, this family of particles will be discussed further in Section 7.

Regarding mRNA, despite their present enthusiasm for therapeutic applications (either for expression of therapeutic proteins [62] or cancer immunotherapy approaches [8,10]), studies on the structures of lipid-mRNA complexes are far scarcer [122,123,124,125]. In general, the behavior of lipid-mRNA assembly tends to follow the behavior observed for DNA, and lipid compositions favoring bilayers with zero curvature tend to favor *L*_α_^C^ structures also in the cases of single stranded RNA [122,123]. However, as pointed out above, mRNA is significantly more flexible than DNA (Figure 1); the charge density is ca. half of that of DNA, which is still highly charged; and the fact that the nucleobases are exposed may lead to additional hydrophobic interactions with the lipids, as it is observed with single-stranded DNA [57,58,59,60]. Cationic liposomes containing DOTAP and 1,2-dioleoyl-sn-glycero-3-phosphocholine (DOPC) were shown to form well-ordered lamellar complexes with mRNA, with SAXS patterns clearly showing the first and second order Bragg peaks [123]. Interestingly, although mRNA has a cross-section radius smaller than DNA, the lamellar spacing (i.e., the lipid bilayer and water slab thickness) of such lipid-mRNA complexes is larger than the equivalent lipid-DNA system [79], and furthermore, the lamellar spacing is seen to increase even further for higher packaging of mRNA (towards ρ_chg_ < 1). Hence, despite being generally similar to lipid-DNA systems, complexes with mRNA also show unique aspects that are important to elucidate towards the design of more efficient systems. It is worth noting that closely related systems, without surface functionalization and just by manipulation of the lipoplexes’ surface charge, have shown promising in vivo activity for anti-cancer vaccines [8,15].

Given the close physical resemblance of mRNA and single-stranded DNA (ssDNA), some of the structures found for lipid-ssDNA and lipid—oligodeoxynucleotide (ODN) systems may also provide additional insights into expectable structural properties of mRNA complexes. As expected, in general, bilayer-forming lipids with *H*_0_ = 0 tend to form also *L*_α_^C^ complexes when complexed with ssDNA and ODNs. This has been convincingly demonstrated for lipid-ODN systems by means of cryo-TEM [126] and SAXS [127]. Interestingly, cryo-TEM shows that ODN-DOTAP lipoplexes have more regular morphologies when compared with DNA lipoplexes. A similar study using two anionic polymers with different rigidity (PAA, with *l*_p_~1 nm and PSS, with *l*_p_~10 nm) showed that besides both systems being lamellar, PAA-DOTAP lipoplexes have a more regular morphology than PSS-DOTAP [128]. These observations may be relevant for mRNA systems, whose *l*_p_~2 nm (Figure 1). Returning to ODNs, it has also been suggested that in cases where the ODN is small, it can induce the formation of *L*_α_^C^ complexes even when the lipid (in this case, a single-tailed surfactant—CTAB) has a strong positive curvature that would not favor the formation of bilayers otherwise [58]. This was the case for a 20-mer made of Adenine (poly(dA_20_)) and a 20-mer with all the bases. In contrast, a 20-mer made of Thymine (poly(dT_20_)) did not induce such structural change. This finding suggests base-specific hydrophobic interactions of the nucleobases with the surfactant or lipid parts which can lead to structural changes. It is also worth mentioning that on a similar system, but using significantly longer ssDNA sequences (denatured calf thymus DNA), a cubic phase with Pm3n symmetry is observed, likely resulting from the ordered packing of spherical CTAB micelles held together by the flexible ssDNA polymer [129], as will be discussed in further detail below (Section 4.3.6).

Taken together, this indicates that in analogy with ssODN and ssDNA, mRNA may be expected to form complexes that also maintain the natural curvature of the lipids—especially naturally forming bilayer lipids. However, the higher flexibility and possibility to engage in hydrophobic interactions can lead to differences in the structures found for the analogous lipid-DNA systems. This should motivate further structural studies as eventual differences between DNA and mRNA could imply different mechanisms of interaction with biological systems.

#### 4.3.2. Inverted Hexagonal Complexes—*H*_II_^C^

The inverted hexagonal structure (*H*_II_^C^, Figure 5) is favored by lipid membranes with negative spontaneous membrane curvature *H*_0_ < 0, such as cationic membranes containing DOPE above a critical molar fraction [98]. These structures are highly efficient at transfecting cells in vitro, presumably due to their ease to fuse with the endosomal membrane, facilitating the lipoplex escape into the cytoplasm (cf. Section 6) [98,130,131]. However, in most cases, the *H*_II_^C^ forms in a narrow lipid composition range, which makes it also somewhat more challenging to formulate reproducibly under broader experimental conditions—a challenge that needs to be taken into account for scaling-up. Whereas in *L*_α_^C^ complexes, the structure can regulate itself by adjusting the separation between the DNA rods, ensuring an optimal matching of cationic charge on the membranes to the amount of DNA intercalated between them, in the *H*_II_^C^ complexes this degree of freedom does not exist [30]. The system therefore separates rather easily into two phases with a *H*_II_^C^ typically coexisting with an *L*_α_^C^ phase, as is found in the DOTAP-DOPE system [98]. The *H*_II_^C^ also forms when mixing DNA with cationic membranes that naturally tend to favor bicontinuous cubic phases instead of inverted hexagonals such as monoolein-DOTAP systems [132]. This is because bicontinuous cubic bilayers would force DNA to bend, imposing an energetic penalty that disfavors the cubic and favors inverted hexagonal phases instead. As will be seen below, the bicontinuous cubic phase is recovered again at the expense of the *H*_II_^C^ if siRNA [133] or non-sticky short DNA [132] are used instead of DNA.

The *H*_II_^C^ also forms when CLs with *H*_0_ < 0 are mixed with siRNA [118]. In contrast with DNA complexes, *H*_II_^C^ siRNA complexes exhibited high toxicity and much lower target-specific gene silencing than their lamellar analogues.

As also mentioned above in the context of the *L*_α_^C^ phase, some ionizable lipid nanoparticles encapsulating siRNA or mRNA can form, at least under some conditions, structures that resemble the *H*_II_^C^ phase [124]. Given their more specific nature, this family of particles will be discussed further in Section 7.

Besides using lipids with negative spontaneous curvature, a second pathway that favors the formation of *H*_II_^C^ phases is by decreasing the membrane bending rigidity κ to very low values, close to the thermal energy (*k*_B_*T*). This is achieved by, e.g., adding a low molecular weight cosurfactant, such as hexanol [98]. Membranes with very low κ favor the formation of *H*_II_^C^ phases because the elastic energy penalty of wrapping the DNA becomes low enough to be compensated by the improved electrostatic interactions between the cationic lipids and the DNA. Because large amounts of hexanol are necessary to induce the transition to the *H*_II_^C^ phase, and also because of hexanol’s high solubility in water, this pathway has not been much explored towards practical applications. In a different context, it was also found that high osmotic pressures of ~80 atm can also induce a transition from the *L*_α_^C^ into the *H*_II_^C^ phase [134]. Although these values are about one order of magnitude greater than physiological osmotic pressures, lowering κ also lowers the osmotic pressure required to induce this transition.

#### 4.3.3. Inverse Bicontinuous Cubic Complexes—*Q*_II_^C^

Inverse bicontinuous cubic phases occur in some natural lipid systems, such as monoolein- and phytantriol-water systems [73,135,136,137]. These structures are favored by lipids with positive saddle splay modulus κ_G_, which drives the formation of membranes with negative Gaussian curvature *C*_1_*C*_2_ (with a saddle-like appearance, cf. Figure 2e). Typically, these phases are based on the Ia3d, Pn3m or Im3m space groups, with the lipids arranged on a continuous periodic minimal surface separating two independent continuous water networks. The term “bicontinuous” reflects the fact that both lipid and water domains are continuous in the three spatial dimensions. These systems can be doped with cationic lipids [138], making them amenable for complexation with NAs. However, when complexed with DNA, the cubic phase is destabilized and the system forms an inverted hexagonal or lamellar phase instead [133,139]. This is likely because, in order to provide a good overlap between the cationic lipid membrane and the DNA, the DNA would need to be substantially bent to follow the topology of the 3D water channels, creating an elastic energy penalty. This is reinforced by the study of complexation between short DNA fragments and cationic bicontinuous cubic phases of the gyroid type (space group Ia3d) [132]. Short DNA strands with five base pairs with blunt ends engage in end-to-end stacking destabilizing the cubic phase and forming *H*_II_^C^ complexes instead. However, if the DNA ends are composed by non-sticky overhangs, end-to-end stacking is diminished and a transition to a *Q*_II_^C^ phase is observed. Overall, these findings demonstrate that the large rigidity of DNA destabilizes the bicontinuous cubic structure, favoring inverted hexagonal phases instead, but if the NAs are short enough, the cubic phase can become stable again. Hence, the *Q*_II_^C^ complex structure represents an example where besides the lipid elastic properties, the NA rigidity and length are crucial at determining the final structure of the complex. This type of insight about the physical properties of building blocks (lipids and NAs) and the crucial interactions between them has led to the development of siRNA-lipid bicontinuous cubic complexes [133], since the siRNA small length and no tendency for end-to-end stacking are amenable to fit in the bicontinuous cubic network [96].

siRNA has been shown to form complexes with an inverted bicontinuous cubic structure of the gyroid type (*Q*_II,G_^C^) when mixed with DOTAP-monoolein systems. The bicontinuous cubic structure and inclusion of siRNA has been convincingly demonstrated by a combination of SAXS and dual-color fluorescence colocalization microscopy [133]. In addition, the cubic unit cell dimension is seen to grow from ~11 nm (before) to ~15 nm (after) mixing with siRNA, while keeping the same symmetry, showing a small adjustment of the native lipid curvature to accommodate siRNA within the two water channel networks. Importantly, this structure was found to have promising gene silencing activity while keeping non-specific gene knockout and toxicity relatively low. This enhanced activity has been suggested to result from the propensity of the cubic phase to fuse with the endosomal membrane. Since the fusion of two membranes results in the formation of a pore with local negative Gaussian curvature *C*_1_*C*_2_, these intermediate structures are easier to stabilize due to the natural negative *C*_1_*C*_2_ in the *Q*_II_^C^ phase. The silencing efficiency of siRNA-*Q*_II_^C^ complexes is further enhanced by the inclusion of multivalent lipids such as MVL5 (+5 nominal charge), the justification being that the multivalent lipid increases the membrane charge density (σ_M_), facilitating the attraction between the cubic and the negatively charged endosomal membranes. The combination of Gaussian negative curvature and enhanced σ_M_ leads to the improved gene silencing efficiency [140].

#### 4.3.4. Normal Bicontinuous Cubic Complexes—*Q*_I_^C^

Another type of bicontinuous cubic phase—the normal bicontinuous cubic (*Q*_I_)—can be formed by surfactants or lipids with a spontaneous curvature that is slightly positive, typically between values that would favor the normal hexagonal or the lamellar phase [73,141,142]. In surfactant/lipid systems, the normal bicontinuous cubic usually belongs to the space group Ia3d. In this case, the periodic minimal surface (gyroid) is the water layer, which separates two independent branched micelle networks composed of the surfactant/lipid.

Despite the difficulty of forming inverted bicontinuous cubic phases complexed with DNA (as discussed above), a normal cubic phase with Ia3d symmetry composed of DNA, dodecyltrimethylammonium (DTA^+^) and lecithin, has been convincingly described [143,144] by means of SAXS and phase diagram determinations. In this case, the DNA is complexed beforehand with dodecyltrimethylammonium bromide (DTAB) at ρ_chg_ = 1 and purified without the inorganic counterions. Under these conditions, the DNA-DTA is considered a complex salt, and the absence of the inorganic counterions simplifies the phase diagram determination by reducing the number of component variables to be considered. It is found that the complex salt DNA-DTA forms a normal tetragonal phase [144] and by addition of lecithin, two transitions, first to a bicontinuous cubic phase (Ia3d) and later to a lamellar phase, are found. The identification of the composition of the single-phase cubic phase (without phase separation) ensures that all the components (DNA-DTA, lecithin and water) are embedded within the same structure, while SAXS clearly identifies the phase structure. The fact that the phase occurs in a lipid-surfactant composition range between normal elongated micelles and lamellar phases allows the identification of the bicontinuous cubic as a normal (rather than inverted) phase [143,144,145]. While this normal bicontinuous cubic phase is unlikely to be of therapeutic relevance due to the high solubility of CTA^+^, these findings help improve our understanding of lipid-DNA systems as a whole and highlight the helpfulness of phase diagrams to identify the nature of the structures present and their relative stabilities [144].

#### 4.3.5. Normal Columnar Complexes—*H*_I_^C^ and *S*_I_^C^

Besides the normal bicontinuous cubic phase, DNA has been reported to form also normal hexagonal (*H*_I_^C^) and square (*S*_I_^C^) phases along with related phases of lower symmetry.

*H*_I_^C^ phases were convincingly demonstrated by SAXS, cryo-TEM and phase behavior considerations for mixtures of DOPC with double-tailed lipids (dioleoyl chains) bearing highly charged dendritic headgroups (nominal charge of +8 and +16) [97,146]. The high charge and size of the dendritic headgroups increase the spontaneous curvature of the lipid films into positive values favoring the formation of highly charged, rod-like micelles. These micelles are arranged in a hexagonal lattice with DNA rods located in their interstices in a honeycomb-like lattice (Figure 5) [97]. As the dendritic lipid fraction over DOPC increases, the rod-like micelles are thought to become shorter and more asymmetric due to the higher preponderance of the dendritic lipid high positive curvature. This is thought to be the cause of the deformation of the hexagonal lattice into a structure of lower symmetry as the dendritic lipid fraction increases [146]. Ongoing with the distorted columnar phases, in the presence of brine and cell-culture media, a highly packed neat DNA columnar phase is also observed, forming probably due to depletion forces arising from the dendritic lipid micelles. Importantly, these normal hexagonal phases assembled from dendritic lipids were shown to have an in vitro transfection efficiency as high as the optimized lamellar and inverted hexagonal phases, with the advantage that the high efficiency is less dependent on the lipid charge density and is particularly high in cell cultures that are traditionally difficult to transfect, such as mouse embryonic fibroblasts [97]. The insensitivity of the efficiency on the membrane charge density hints at a different mechanism of transfection, possibility related with the continuous DNA substructure, which likely facilitates the release of DNA once a part of it becomes exposed to the cytosol [97,146].

Normal hexagonal phases with some degree of distortion are also thought to form in single-tailed cationic surfactant-DNA systems [58,129,147,148,149,150,151,152]. Small-angle X-ray and neutron scattering show structures consistent with hexagonal symmetry. While frequently the scattering patterns do not allow a clear distinction between normal and inverted hexagonal phases (see e.g., Ref. [129]), the relative position of these phases on the phase diagrams of surfactant-DNA complex salts (see e.g., Ref. [144]) make them more consistent with normal than inverse structures. This point is further demonstrated through DNA-CTAB-hexanol systems [149,150]. As hexanol acts as a cosurfactant, it decreases the spontaneous curvature of lipids, with CTAB micelles shifting from normal to reverse when enough hexanol is added. Hence, through the addition of hexanol to DNA-CTAB complexes, the scattering patterns transit from a hexagonal at low hexanol, to lamellar at intermediate hexanol, and finally to another hexagonal at high hexanol content [149,150]. This phase evolution is consistent with a continuous decrease of the lipid curvature following the addition of hexanol, which allows the identification of the first phase as a normal hexagonal, and the third phase as a reverse hexagonal. When the inorganic counterion of CTAB (Br^−^) is replaced by tosylate (an organic counterion that binds strongly to the micelles), a normal square phase (*S*_I_^C^) between the surfactant and DNA is found at lower DNA-to-surfactant ratios [152]. This phase becomes viable in this system because the otherwise uncompensated excess charge from the micelles is balanced by the strongly binding tosylate counterions. As mentioned above, the single-tailed surfactant-DNA complexes are not expected to have a high therapeutic relevance due to the high solubility (and toxicity) of the surfactants.

The normal hexagonal has also been reported for complexes of DNA with cationic gemini surfactants [153]. Cationic gemini surfactants are a type of surfactant constituted by two single-tailed cationic surfactants covalently linked by a molecular chain of variable length (called “spacer”). These surfactants can have high positive curvatures that favor the formation of normal micelles. Such micelles, formed from butane-1,4-diyl-bis(alkyldimethylammonium bromide) gemini surfactants were found to complex with DNA, forming *H*_I_^C^ phases for surfactant tail lengths of 12, 13 and 14 carbons [153]. Such normal hexagonal gemini—DNA complexes were found to have poor transfection efficiency, in contrast to lamellar complexes resulting from the combination of gemini with neutral lipids, which were found to be more promising [154,155].

#### 4.3.6. Other Normal Phases

Single-tailed cationic surfactants (e.g., CTAB and DTAB) tend to form spherical micelles in the absence of DNA. The formation of columnar phases with DNA (as discussed above) in which the surfactant is likely arranged in a rod-like micelle results from the high-rigidity of DNA. If long single-stranded DNA is used instead, the hexagonal phase can be replaced by a normal discrete cubic phase (*I*_I_^C^), presumably because now the much higher flexibility of ssDNA is able to accommodate the natural curvature of the cationic surfactant [129]. This and related structures are also frequently observed in other flexible polyelectrolyte-surfactant systems [156,157,158]. Alternatively, as already pointed out above, if the ssDNA is very short, it can induce the formation of lamellar phases instead, in a base-sequence specific manner, likely due to hydrophobic interactions between the bases and the surfactant [58].

Some cationic gemini surfactants forming normal micelles were found to complex with siRNA and seem to form a type of condensed micellar phase with siRNA sandwiched between gemini surfactant micelles [159]. In this study, the assembly time was restricted to 15min after mixing between siRNA and the surfactant since it is the time needed for complex formation and administration into biological systems. It is possible that at longer times (hours/days) these complexes could arrange into ordered phases like the ones discussed above (e.g., normal cubic or normal hexagonal). In a related gemini surfactant family, composed of bis-imidazolium gemini surfactants, although a condensed micellar phase in the presence of siRNA was also the most prevalent structure, in some cases, depending on the spacer length and charge ratio ρ_chg_, additional Bragg peaks in the scattering patterns were consistent with more ordered structures, possibly hexagonal and cubic [160].

## 5. PEGylation—Improved Circulation Lifetime and Effects on the Particle Structure

In the previous section, we discussed the internal structures/symmetries of lipid-NA systems. Importantly, these different structures interact with cells via different mechanisms (c.f. Section 6) and can be optimized for suitable transfection efficiencies in vitro. However, most of these systems as described up to now are not suitable for systemic applications in vivo as they are rapidly removed from circulation. Coating lipid-NA systems with a PEG layer was found to sterically stabilize lipoplexes and significantly improve their circulation lifetime. The addition of PEG also induces structural changes in the particles, either at the level of their size or internal structure/morphology. In this section, we will discuss some of these aspects.

### 5.1. PEGylation—Improved Colloidal Stability and Circulation Lifetime by Steric Stabilization

Although there are exceptions [8], most of the lipid-NA systems outlined above are not suitable for systemic applications. Cationic lipids and their nucleic acid complexes activate the complement system [161], resulting in their rapid removal from circulation by the mononuclear phagocytic system [78]. By acknowledging that red blood cells are not removed by the phagocytes in the bloodstream, the first strategy in trying to overcome this barrier was to mimic the red cell membranes by incorporating the sterically hindered ganglioside GM_1_ or phosphatidylinositol (PI) in the liposomal membrane. This led to a significant improvement in the circulation time of these now-called “stealth” liposomes [162,163]. Subsequently, it was realized that other flexible-chained hydrophilic polymers were also successful at improving the circulation time of liposomes in blood. Namely, polyethylene glycol-lipids (PEG-lipids), with PEG molecular weights above 1900 Da, provided longer circulation times compared to GM_1_ and PI [164,165,166]. Thus, together with their availability, PEG-lipids became the main choice for steric stabilization. This stabilization is mainly entropic in origin [167,168]. As colloidal objects approach the PEG coating, the PEG chains become confined, leading to a reduction of the entropy. This produces a repulsive force that decreases the attachment of blood opsonins as well as other elements, thus leading to a reduction in complement activation. The magnitude of the repulsive force depends on the amount of PEG on the liposome surface. In the so-called brush regime, that is, when the average distance between neighboring PEG chains is smaller than the PEG radius of gyration, the surface coverage is nearly full, leading to an expansion of the polymer outwards of the membrane to minimize the overlapping of the chains [167,168]. This regime, which for PEG2000 (M_w_ ≈ 2000) occurs for a PEG-lipid molar fraction of ~10% [117], is most efficient for steric stabilization.

Cationic lipid membranes incorporating PEG2000-lipids successfully associate with DNA leading to the formation of PEGylated lipid-NA nanoparticles with *L*_α_^C^ structure [169]. Inclusion of the PEG layer in the lipid-DNA particles leads to improved colloidal stability, preventing particle aggregation and flocculation in physiological ionic strength [93,169]. Importantly, these complexes also show prolonged circulation lifetime and are suitable for in vivo studies [170,171]. Alternatively, PEG-lipids can be post-inserted into lipid-NA particles with similar efficiency [172,173,174].

Another significant benefit from PEGylation is the ability to use the PEG chains to tether specific ligands to direct lipid-NA particles to specific targeted cells [171,175], while simultaneously the PEG brush helps shielding off the cationic charge to decrease non-specific binding to off-target tissues [93].

One significant drawback from PEGylation is that this same PEG surface coating that provides improved circulation lifetime also decreases significantly the ability of the lipoplexes to fuse with the endosomal membrane and escape, in what is commonly known as “the PEG dilemma” [17,176]. Fortunately, some clever strategies exist to remove PEG from the particles before it becomes detrimental. Examples of such strategies are, for instance: the inclusion of acid-labile bonds between the PEG and alkyl tail moieties, designed to cleave inside late endosomes when the pH drops and facilitate endosomal release [177,178]; or use of diffusible PEG-lipids, which use shorter alkyl tail moieties that lead to a gradual loss of the PEG coating after systemic administration [179]. Another concern is that in some cases, after multiple dosing, PEG may lead to immune responses [26].

### 5.2. Size and Structure Modulation In Pre-Formed Lipid Assemblies

Besides improved circulation times, PEGylation also impacts the structure of the particles. Depending on the assembly route and solvent conditions, the amount of PEG-lipid can determine the size and the structure of the particles in multiple ways [117,169,180,181].

When preformed PEGylated liposomes are used to make lipoplexes, it has been observed that the formed particles decrease in size and become colloidally more stable even in cell culture media [93,169]. Interestingly, it was also observed that the distance between the DNA molecules sandwiched between lipid bilayers in the *L*_α_^C^ phase becomes smaller. This occurs because, when using preformed PEGylated liposomes, the PEG anchored on the inner lipid bilayers has to compete with DNA for the same water volume, pushing DNA chains against each other [169].

**Figure 6:**
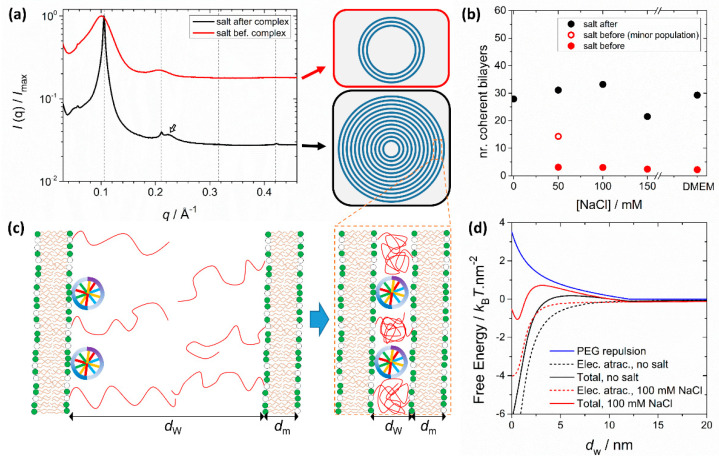
PEGylation modulates the number of lamellar layers of lipid-DNA complexes depending on the media salt concentration. (**a**) SAXS patterns for PEGylated lipid-DNA complexes at ρ_chg_ = 3 and [NaCl] = 100 mM. The liposome composition is the same for both systems (80/10/10 for DOTAP/DOPC/DOPE-PEG2000 in molar fraction). The SAXS pattern in black results from complexes prepared in water and transferred to brine later. The SAXS pattern in red results from complexes prepared in brine from the beginning. The dashed lines indicate the positions of the expected *L*_α_^C^ Bragg peaks, with a *d* spacing (*d*_m_ + *d*_w_) of 5.97 nm. Complexes prepared in water show relatively narrow Bragg peaks, indicative of large domains containing a large number of coherent layers. Complexes prepared in salt have significantly broader peaks, indicative of smaller lamellar domains, with a low number of coherent layers. The hollow arrow indicates the DNA in-plane spacing (ca. 2.82 nm) peak, visible for complexes prepared in water. (**b**) Analysis of the line shape of the Bragg peaks allows estimating the number of coherent lamellar layers in each complex. The differences between both pathways (salt before or after complexation) are more evident with increasing amounts of salt. (**c**) Schematic representation of the complexation process between one lipid bilayer overcharged with DNA (hence, overall anionic) and a cationic lipid bilayer. Whereas electrostatic forces yield an attractive force and favor complexation, compression of the PEG polymers results in a repulsion force that needs to be overcome. (**d**) Calculated free energy contributions of the electrostatic attraction and repulsive force from PEG compression as a function of the membrane distance and for different amounts of salt. In the presence of salt, the electrostatic attraction is reduced. While the global energy minimum still resides at very small membrane separations (complexed state), the emergence of an energy barrier means that some complexes will be kinetically locked with a smaller number of layers. The data shown is replotted from Ref. [117].

PEGylation can also significantly influence structure depending on the assembly route and solvent conditions. It was shown that preformed PEGylated liposomes interact less strongly with DNA when forming complexes, making the complexation process more sensitive to the ionic strength of the media [117] (Figure 6). This is because the favorable Coulomb interactions between the opposite charges of liposomes and DNA and entropy gain due to counterion release are now counteracted by the unfavorable confinement of the PEG moieties to the water interstices of the *L*_α_^C^ (Figure 6c). When formed in water, the electrostatic attraction dominates the steric repulsion from PEG and SAXS measurements show that these complexes form with a large number of lamellar layers (Figure 6a). When transferred to saline media, the complexes remain stable due to the steric stabilization of the PEG layer outside, and because the *L*_α_^C^ is still favored thermodynamically. However, if the PEGylated liposomes and DNA are mixed already in saline media, the electrostatic attraction is significantly decreased by the screening effect of salt and becomes comparable to the steric repulsive force from PEG. Under these conditions, *L*_α_^C^ complexes still form but now with a much smaller number of lamellar layers (Figure 6b). The lamellar structure is still the thermodynamically preferred state, but the complexes become kinetically locked with a smaller number of layers than the ones formed in water. This behavior is modulated by the amount of PEG, lipid membrane charge density and media ionic strength [117]. These findings show that small differences in the preparation protocol result in significant differences in the morphology of PEGylated cationic lipid-DNA complexes. On one hand, this recommends caution when comparing transfection data across different laboratories. On the other, it provides another means to further manipulate the structures of lipoplexes.

Bicontinuous cubic phases containing siRNA (*Q*_II_^C^) can also be PEGylated. Besides improved colloidal stability and smaller sizes, it was observed that the inclusion of monoolein-PEG also induces the formation of two cubic phases in the absence of siRNA: a gyroid (space group Ia3d) and a primitive (Im3m) bicontinuous cubic phase. Interestingly, after complexing with siRNA, it is the primitive cubic that prevails [182]. These complexes, nicknamed “cuboplexes”, show promising siRNA delivery and specific gene silencing. Whereas mixing preformed dispersed cubic phases (cubosomes) with siRNA resulted in particles with sizes of ca. 300 nm, a microfluidic device employing a solvent-exchange mixing scheme was able to produce smaller particles (~75 nm) [183].

### 5.3. Solvent-Exchange and Monomolecular Nucleic Acid Lipid Particles

When forming lipid-NA complexes via solvent-exchange (without pre-formed liposomes), the amount of PEG can also be used to limit the growth of the particles during annealing or while exchanging ethanol with the final buffer [121,180,181]. Solvent-exchange approaches typically imply the dissolution of the lipid mixture in ethanol, which is subsequently mixed with an equal volume of NAs dissolved in an aqueous buffer. The mixing between ethanol and water leads to a drop in the solubility of the lipid, inducing the formation of macromolecular assemblies. After mixing, the high amounts of remaining ethanol can lead to substantial rearrangements, which depend also on the solubility of PEG-lipids. These conditions can be manipulated to favor the emergence of well-structured lamellar layers, or to lock the particles in more disordered structures [181]. Importantly, because of the extra mobility of the PEG-lipids in the high-ethanol solution, these tend to accumulate at the particles’ surface, thus effectively limiting particle growth by fusion [121]. Hence, the amount of PEG can be used to control the particle size [180]. This approach is frequently implemented using microfluidic devices to control the assembly process with greater precision [180,184,185]. Some of these aspects will be discussed in Section 7 in the context of ionizable lipids.

A solvent-exchange assembly scheme, either performed using more traditional mixing methods [186] or using microfluidic mixing [175] was also used to prepare monomolecular nucleic acid lipid nanoparticles, which are arguably the smallest lipid-based NA carriers. The encapsulation of single siRNA molecules in lipidic particles was shown to be possible if the lipid components can accommodate an asymmetric lipid bilayer, in which the inner membrane leaflet is cationic and with an inverted curvature to engulf siRNA (achieved with e.g., DOTAP and DOPE) and the outer leaflet has normal curvature and a PEG coat to sterically stabilize particles against aggregation [175,186]. This asymmetric cationic-zwitterionic PEGylated lipid bilayer can form stable, monodisperse, and approximately 30 nm in size particles. These PEGylated siRNA constructs are capable of protecting the NAs against nucleases and, as a result of high steric stability, avoid particle aggregation and nonspecific binding and opsonization by blood proteins of the complement system [186].

## 6. Particle Structure, Charge and Functionalization Influence on the Transfection Efficiency

In the previous sections, we have discussed different structures of lipid-NA particles and how their physical properties can be influenced by parameters such as cationic-to-anionic charge ratio (ρ_chg_), membrane charge density (σ_M_) and PEGylation. In this section, we briefly discuss how these variables influence the interaction of lipid-NAs with cells in the expression/silencing of genes.

### 6.1. Cell Uptake: Nonspecific Electrostatic Interactions and Targeting with Affinity Ligands

Lipid-NA particles tend to be internalized by cells via endocytosis [4,19,20,25,187]. As these particles are usually prepared with excess cationic lipid (ρ_chg_ > 1), they have an overall net-positive charge that mediates attractive electrostatic interactions with the anionic proteoglycans from the cells surface. This favors nonspecific cell attachment and subsequently endocytosis. Besides the net-positive charge, nonspecific cell attachment is also favored by high σ_M_, since it leads to a larger electrostatic attraction [93].

PEGylation, which is important for in vivo gene delivery, reduces cell uptake to some extent as a result of a weaker electrostatic attraction of the lipid-NA complexes to the cell membranes. However, cellular uptake in PEGylated particles can be recovered by attaching targeting ligands to the distal ends of PEG, which allows for targeted delivery and receptor-mediated endocytosis [25,188]. For example, when using PEGylated particles tagged with an RGD peptide (which binds to the α_5_β_1_ integrins to initiate receptor-mediated endocytosis), both low and high σ_M_ PEGylated particles showed a marked increase in particle uptake when compared to identical particles without the peptide. Among the RGD-tagged particles, those with high σ_M_ show a marginal improvement on uptake when compared to low σ_M_, which demonstrates that even though PEG strongly attenuates the effect of charge, at high σ_M_ nonspecific electrostatic interactions are not fully eliminated [93]. These residual nonspecific interactions can become critical in vivo and interfere with the specific targeting desired by the ligands [171]. By using a broader collection of peptide-tags, it was also shown that while for positively charged particles (ρ_chg_ > 1) the effect of charge on peptide-tagged particle uptake is mild, particles with negative charge (ρ_chg_ < 1) showed no cell uptake even with peptide tags. Importantly, it was also shown that an intermediate level of particle surface coverage with RGD led to better uptake than low or high RGD coverage [171].

In contrast, in vivo studies on mice have shown that simple cationic lipid-mRNA lipoplexes without PEG nor targeting ligands underwent enhanced accumulation in the spleen when their net charge was negative (ρ_chg_ < 1). This led to enhanced targeting of dendritic cells and stimulation of the immune system against cancer, leading also to promising results in early-stage clinical trials [8].

### 6.2. Endosomal Escape and Transfection Efficiency: Lipid Membrane Curvature and Charge Density

After internalization, lipid-NA particles become trapped inside endosomes, being trafficked through different endocytic paths that may end up in lysosomes leading to their destruction, or in recycling paths that take them back to the cell exterior [189,190]. Hence, in order to exert their therapeutic activity, lipid-NA particles have to escape from the endosome. Endosomal escape is one of the major bottlenecks in achieving high transfection.

Efficient endosomal release seems to be related with the ability of the lipid-NA particles to fuse with (or destabilize) the endosomal membrane, opening a pore from which they can escape [130]. Enhanced fusion with the endosomal membrane can be achieved through two main ways: (i) lowering the elastic energy penalty of the endosomal and lipid-NA membranes’ deformation when they fuse, and (ii) increasing the electrostatic attraction between the particle and the endosomal membrane [78].

Fusion with the endosomal membrane involves the formation of intermediate structures with negative curvature (*H* < 0), resembling inverted hexagonal phases [191], and the formation of a pore across two bilayers with local negative Gaussian curvature (*C*_1_*C*_2_ < 0, Figure 2e) [140]. This membrane deformation has an elastic energy cost for normal bilayers, but lipid-NA phases that already have *H* < 0, such as the *H*_II_^C^ phase [98,130,192] or positive saddle splay modulus κ_G_ (which favors *C*_1_*C*_2_ < 0), such as the *Q*_II_^C^ [133,140], will have a significantly smaller energetic barrier to form such intermediate structures and fuse more easily with the endosomal membrane. The *H*_II_^C^ and *Q*_II_^C^ phases show indeed high transfection/silencing efficiency [130,133]. The ability for bilayers to transition to an inverted hexagonal phase has also been used as a predictor for membrane fusion and endosomal escape [131].

**Figure 7:**
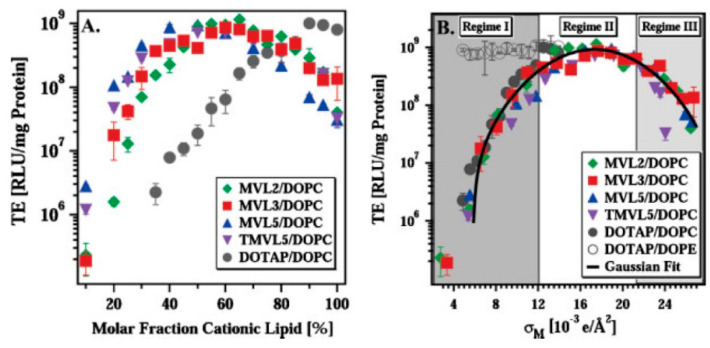
Transfection efficiency (TE) of *L*_α_^C^ complexes versus cationic lipid molar fraction and membrane charge density (σ_M_). The ρ_chg_ is fixed at 2.8 (**A**) The TE increases with the cationic lipid molar fraction, reaching a peak before decreasing again. The TE peak moves to smaller cationic fractions for larger cationic lipid nominal charge. (**B**) When the TE is replotted as a function of σ_M_, the data for the different headgroup charges (+1 to +5) all collapse into a universal bell-shaped curve that can be sub-divided into three regimes. Regime II has a TE comparable to the TE of *H*_II_^C^ complexes. Reproduced with permission from [193]. Copyright 2005 John Wiley & Sons Limited.

The other critical factor influencing the endosomal release and consequently the transfection and/or silencing efficiency is the electrostatic attraction between the lipid-NA particles and the endosomal membranes. This is especially relevant for *L*_α_^C^ lipid-NA complexes whose elastic energy of membrane deformation is expected to be higher than in the *H*_II_^C^ and *Q*_II_^C^ phases. By keeping the hydrophobic moieties of the bilayers constant, and by gradually adjusting the fraction of cationic over neutral lipids for fixed ρ_chg_ = 2.8, it was observed that the transfection efficiency (TE) increased gradually for larger cationic lipid mole fractions [130] (Figure 7). Importantly, when using multivalent cationic lipids (MVLs) such as DOSPA or MVL2-MVL5 (nominal charge from +2 to +5), the TE curve changes shape, now showing a rapid TE increase followed by a decrease as a function of the cationic lipid molar fraction [130,193]. The TE peak moves to lower cationic lipid molar fractions as the nominal charge of the multivalent lipid increases (Figure 7a). Remarkably, if the TE is replotted now as a function of the membrane charge density σ_M_, the curves for the different headgroups all collapse into a universal bell-shaped curve [193] (Figure 7b). This clearly demonstrates that all other factors being equal (e.g., ρ_chg_, neutral lipid and cell line), the σ_M_ is a universal parameter determining the transfection efficiency in vitro. This is remarkable and reveals fundamental aspects about the interactions of lipoplexes with cells. This knowledge should be translatable to in vivo, provided that the complexes reach the cells.

Combined TE measurements and live-cell imaging allow ascribing the large impact of σ_M_ on the TE mainly to an enhanced endosomal release and, to a lesser extent, to a slightly better cellular uptake [93,190]. The bell-shaped curve shows three distinct regimes (Figure 7b). In regime I, the TE increases by more than three orders of magnitude going from low to higher σ_M_. As the σ_M_ increases, the electrostatic attractions between lipid-NA particles and the endosomal membrane also increase, leading to more fusion events, superior endosomal escape and consequently higher TE. For very high σ_M_ (regime III), a decrease in the TE is observed with increasing σ_M_, suggesting that there is also an obstacle of electrostatic nature to successful NA delivery [193]. One hypothesis for this obstacle could be the formation of very stable lipid-NA interactions at such high σ_M_, preventing NA release from the complex even after escaping the endosomes. Regime II is likely to result from the overlap between regimes I and III, and represents the optimal σ_M_ range for enhanced TE. Notably, this regime II shows a level of TE similar to the TE of inverted hexagonal phases (Figure 7b), demonstrating how knowledge of the particle structure and the crucial interactions with biological systems helps to optimize the TE. This also clearly demonstrates the importance of neutral lipids in the formulations of lamellar lipid-NA particles, especially when including multivalent lipids [193].

Even though the inclusion of multivalent lipids per se does not augment substantially the TE due to the saturation of regime II, it is still advantageous since the same optimal σ_M_ can be achieved with a significantly lower amount of cationic lipid molecules and hence decrease cytotoxicity. MVLs are also important for siRNA delivery, which requires large ρ_chg_ [3,118]. Indeed, MVL5 siRNA *Q*_II_^C^ complexes show remarkably high silencing efficiency due to the combination of the enhanced electrostatic attractions (from the high σ_M_ imposed by MVL5) with the Gaussian negative curvature stabilization from the *Q*_II_^C^ phase [140].

Lipid-NA particles with the normal hexagonal structure (*H*_I_^C^) assembled from dendritic lipids were also shown to have an in vitro transfection efficiency as high as the optimized lamellar and inverted hexagonal phases. Their mechanism of action, however, is still unresolved [97].

Unfortunately, when lamellar lipid-NA particles are PEGylated, endosomal release becomes seriously hampered. This results from the steric repulsion imposed by the PEG brush, which prevents the particle membranes from coming close enough to the endosomal membrane and form a pore across the two bilayers [93,169,178]. As will be seen below, endosomal release can be recovered by using acid-labile PEG lipids [177,178] or diffusible PEG lipids [179].

Further detail about the specific endocytic pathways that particles can take and how trafficking is influenced by the complexes’ biophysical properties (e.g., size, charge and targeting ligands), can be obtained by live-cell imaging methods. While a thorough description is outside of the scope of this work, the interested reader can find more details in the available literature [189,190,194,195,196].

### 6.3. Exploring Intracellular Stimuli

We mentioned at the end of Section 5.1 and Section 6.2 that the detrimental effect of PEG on the transfection efficiency can be overcome by the inclusion of acid-labile linkers between the PEG and lipid backbone, which cleave when the pH drops in late endosomes [17,177]. The inclusion of PEG lipids with an acid-labile acylhydrazone moiety between the lipid and PEG backbones sterically stabilized lipid-NA NPs in a manner similar to conventional PEG-lipids at pH 7.4 for 24h, but when transferred to pH 5, the particles became unstable. Importantly, the in vitro transfection efficiency was shown to reach values comparable to non-PEGylated particles. The observation that conventional PEG and acid-labile PEG lipid-NA particles distribute equally inside cells (likely inside endosomes) is strong evidence that the enhanced TE is caused by the detachment of PEG in the late endosomes, which facilitates endosomal release and recovery of TE to values closer to non-PEGylated particles [178]. The drop in pH in the late endosomes can also be explored to increase the charge density of particles containing ionizable cationic lipids, as discussed in the next section.

Another intracellular stimulus that can be exploited to improve the efficiency and lower the toxicity of lipid-DNA particles is the high-reducing environment inside the cells, which can cleave disulfide bonds [17]. This can be used to promote the disassembly of the particles and simultaneously reduce the toxicity of the cationic lipids. A series of multivalent headgroup lipids with a disulfide link between the headgroup and alkyl tails was synthesized leading to stimuli-responsive particles that are stable in oxidizing media but irreversibly change morphology as the media becomes reducing, indicative of the disulfide linker cleavage [197,198]. Importantly these particles show high transfection efficiency, similar to their non-cleavable headgroup analogues and common lipofectamine benchmark, but with the advantage of showing significantly reduced toxicity [197].

## 7. Ionizable Cationic Lipids and Lipid Nanoparticles (LNPs)

Therapeutic strategies utilizing cationic lipids have been promising even for simple non-functionalized lipoplexes, which have shown recent exciting results in early-stage clinical studies [8]. In many instances, however, issues related to internalization into tumor cells, cytotoxicity, immunogenicity and short half-life in circulation as a result of nonspecific binding to serum proteins [199,200] have led to a search for more alternatives. A clever approach to overcome the dilemma of the requirement of charge for endosomal release and nonspecific binding resulting in a low biodistribution utilizes ionizable cationic lipids [201,202]. These ionizable lipid-like materials reduce the toxic side effects of lipoplexes but maintain their transfection characteristics [14,26,203]. These formulations containing an ionizable cationic lipid core have gained considerable interest, in particular with the recent FDA approval of the first non-viral gene therapy medicine (for the treatment of hereditary transthyretin-mediated amyloidosis) [19].

Typically, ionizable lipids have been formulated together with three more components: PEGylated lipid, to increase circulation half-life in vivo, control particle size and avoid nonspecific uptake; cholesterol, to increase lipid bilayer stability; and a naturally occurring helper phospholipid to support the lipid bilayer structure [204]. These particles are commonly designated by lipid nanoparticles (LNPs), although strictly speaking they are also lipoplex systems.

Unlike traditional lipoplexes, which tend to be assembled from pre-formed liposomes mixed with NAs in aqueous buffer, LNPs are now typically produced by solvent-exchange approaches using microfluidic methods [175,180,205] or confined volume mixing platforms [181] for fast mixing. Under these conditions, and by controlling the amount of PEGylated lipids in the formulation, LNPs can be made with relatively small sizes of ~30–100 nm and low polydispersity, making them ideal for clinical translation [19]. These LNPs were shown to form through a fusion-dependent process, where, as a result of the ionizable lipid pH neutralization step, smaller particles fuse until the final particle accumulates sufficient PEG-lipid on the surface to sterically inhibit further growth [206]. The size is therefore controlled, to a large extent, by the amount of PEGylated lipid [121,124,180] (c.f Section 5.3).

Series of ionizable or pH sensitive cationic lipids have been developed with acid dissociation constant (pKa) values of 7 or lower [131,207,208,209]. This means that in environments where the pH is below the pKa, the ionizable lipids are positively charged, promoting association with negatively charged NAs. When the pH of the medium is changed to physiological, the surface of the LNPs becomes almost neutral in charge. This makes them suitable for systematic administration avoiding the demerits of the permanent charge as with the conventional cationic lipids [14,210]. An additional relevant feature is the ability to bind to endogenous proteins in circulation, like apolipoprotein E (ApoE), which efficiently mediates the delivery to hepatocytes via the low-density lipoprotein (LDL)-receptor pathways [211]. The pKa of the ionizable cationic lipids is a critical parameter in order to obtain potent formulations. The pKa has to be low enough to prevent the LNP from having a high surface charge at physiological pH, which would lead to rapid clearance from circulation and toxicity. However, the pKa should be high enough so that the ionizable lipid can build up positive charges, critical in destabilizing the endosomal membrane [212]. Once taken up by the cells, these LNPs build up positive charge in acidic environments, such as endosomes, which reduce their pH from ~6.8 to 4.5 as they transition to lysosomes. The build-up in positive charge is critical for aiding in endosomal escape and intracellular NA delivery. Although the precise mechanism of endosomal release remains unclear, it is suggested that the acquired positive charge of the LNPs enables binding to negatively charged lipids in the endosomal membranes. This can induce fusion or pore formation via intermediate non-bilayer structures such as the hexagonal *H*_II_^C^ phase, which can facilitate endosomal release [98,130,131,192,213]. A related design parameter of importance is that the cationic lipid should be relatively unsaturated as it is established that these lipids adopt more readily non-bilayer structures [212].

The structures of ionizable lipid-NA assemblies can be somewhat elusive. One of the keys to its rationalization is acknowledging that the ionizable lipid has a different curvature when in the ionized or neutral forms. This leads to a dependence of the particle structure not only on the lipid composition, but also on the ρ_chg_, as explained below. Ionizable lipid mixtures containing DSPC, cholesterol and PEGylated lipids form liposomes at pH 4 (when the ionizable lipid is in the cationic form) and form liposomes with an electron-dense amorphous core at pH 7.4 (when the ionizable lipid is neutral) [121]. This indicates that the curvature of the ionizable lipid becomes significantly more negative in the neutral form. While ionized cationic lipids complexed with NAs are probably less likely to be affected by pH changes, any excess of ionizable lipid (i.e., at cationic-anionic charge ratios ρ_chg_ > 1) is expected to influence the structure in the same way as adding a negative curvature helper lipid (i.e., DOPE). Hence, the structures of LNPs containing ionizable cationic lipids depends also on the cationic-anionic charge ratios ρ_chg_ (see e.g., Ref. [121]).

For high ρ_chg_ (assuming full lipid ionization) siRNA LNP systems exhibit an electron-dense hydrophobic core as visualized by cryo-EM and in agreement with NMR experiments and molecular modeling [184]. This led initially to suggestions that the inner structure of LNPs consists of inverted micelles (L_2_) of lipid encapsulating siRNA surrounded by a coating of PEG-lipids [184]. Later experiments suggest a slight reinterpretation—that the electron-dense hydrophobic core is mostly composed of neutralized ionizable lipid in excess that, along with some cholesterol, segregates into the particle core [121]. The siRNA, instead of being in the core is now sandwiched between this core (which is ionized at the surface in contact with siRNA) and an outer lipid bilayer containing ionizable cationic lipid, DSPC and PEG-lipid [121]. In contrast, similar formulations using mRNA instead of siRNA seem to indicate that the inner structure seems to be indeed of the inverted type with mRNA and some water uniformly distributed across the particle. In this case, however, perhaps because of the long mRNA compared to siRNA, the structure resembles more a disordered inverted hexagonal phase than inverted micelles [124] (see below).

For ρ_chg_ ~1, that is, at higher siRNA amounts approaching the encapsulation limit, the structure of LNPs resembles more the traditional *L*_α_^C^ lamellar complexes described above [119,121] (Figure 5). Because the hydrophobic core observed at high ρ_chg_ is composed of excess ionizable lipid, the fact that most ionizable lipid becomes involved in the complexation with siRNA at ρ_chg_ ~1 (and hence, less susceptible to pH changes) is likely to be the reason for the observed structural change [119,121]. Hence, not only does the stimuli-responsive nature of the lipid influence the surface properties of the particles, being one of the reasons suggested for their enhanced efficiency, but also they add complexity into the structural behavior of the formulations.

Another important factor is the amount of PEG-lipid and its relation to the annealing time and solvents used [181]. SAXS data showed that besides controlling the LNP size, larger amounts of PEG-lipid anchored on the particles also hinder internal structural rearrangements of the lipids, independently of particle size. The amount of lipid anchored on the particles is not only a function of the PEG-lipid nominal concentration, but also of the solvents used. The relatively high amounts of ethanol used in the solvent exchange approach increase the solubility of PEG-lipids, which result in a smaller number of PEG-lipids anchored on the particles during the annealing time before ethanol is reduced and pH raised. By controlling the quality of the solvent for PEG, the annealing time and nominal PEG-concentration, the LNP structure can be lamellar or more disordered [181].

As pointed out above, the structures of mRNA-based ionizable lipid LNPs with ρ_chg_ = 3 seem to be more consistent with disordered inverted hexagonals engulfing the mRNA. By means of small-angle neutron scattering (SANS) utilizing selective contrast variation of lipids and solvent, it was convincingly shown that the core contained ca. 24% of water and mRNA uniformly distributed along the particle [124]. Importantly, and as hinted in other studies, it was also confirmed that DSPC is mostly located on the surface of the LNPs. Furthermore, when measuring the structure of bulk lipid-mRNA compositions identical to the perceived composition of the LNPs core, the SAXS scattering patterns show an inverted hexagonal structure at pH 3. At physiological pH the higher order peaks vanish, but the location of the first peak remains the same. The position of this first peak is also the same as in LNPs, which suggest that the internal structure of LNPs is that of a disordered inverted hexagonal phase [124]. The disorder is likely to be enhanced by the neutralization of the excess ionizable lipid at higher pH, which has a lower curvature than the ionized form, as discussed above. The determination of the internal structure and the insight that DSPC is located preferentially on the LNP surface provides important hints to optimize the TE. In particular, it was found that a surface area per DSPC molecule of 1.2 nm^2^ (which is more than twice the area needed for a DSPC molecule) gave rise to optimal in vitro transfection, whereas lower areas per DSPC provided lower transfection efficiencies [124]. This lower occupancy of the LNP surface by DSPC can be important to let ionizable lipid and cholesterol be part of the surface. In the acidic environment, the ionizable lipid is likely to become positively charged, leading to favorable interactions with the negatively charged endosomal membranes and lead to endosomal escape. The area per DSPC may carry then a tendency similar to the TE versus σ_M_ curve shown in Figure 7b. The solubility of cholesterol in the LNP core was also found to be relatively low which can lead to the accumulation of cholesterol on the surface [124]. This can also impact the TE [214].

## 8. Targeting the Tumor

We have seen above how particle functionalization with PEG can be used to improve the circulation lifetime of lipid-NA complexes, and how intracellular stimuli can be used to improve the transfection efficiency. Tumors also have a series of special characteristics that can be exploited for improved targeting. This targeting can be passive, exploiting the tumor leaky vasculature, or active, in which targeting ligands are added to the particle to recognize overexpressed extracellular biomarkers in the tumor or tumor microenvironment. A comprehensive description of these characteristics and targeting strategies is outside of the scope of this work as a significant number of excellent reviews are already available to the interested reader [188,215,216,217,218]. Here, we will restrict to a couple of examples that we find particularly important and general.

### 8.1. Passive Targeting

The Enhanced Permeability and Retention (EPR) effect is a phenomenon that leads to the enhanced accumulation and retention of macromolecules and nanoparticles in solid tumors compared to normal tissues [188,219]. This improved accumulation results from the tumor vasculature that grows abnormally in order to feed the rapidly growing tumor. This results in leaks in the vasculature, from which nanomedicines can penetrate the tumor. However, the EPR effect is not a universal property of all solid tumors and care should be taken when exploring this route of passive tumor targeting [219]. Some tumors have a very dense extracellular matrix with very limited EPR-based accumulation (e.g., pancreatic ductal carcinoma). In addition, analysis from 200 different patient tumors across eight different cancer types have revealed marked differences in the tumor vasculature and morphology, observed between tumor types, between tumors of the same type, and even within the same tumor [218,219]. Despite this large heterogeneity, a good number of patients could still benefit from EPR-based passive targeting, and improvements in diagnostics to identify which patients could benefit from EPR would lead to better treatment outcomes [220]. To make use of the EPR effect, PEGylation is commonly used to increase the circulation lifetime long enough to allow the accumulation of the therapeutic nanoparticles in the tumor. As described above, PEGylation can also be used to achieve tunable sizes in the 30-100 nm range [124,180], and this size tunability could be employed to improve perfusion in denser tumors.

### 8.2. Active Targeting

To minimize off-target effects, the accumulation in tumors can also be enhanced by affinity ligands (e.g., peptides, antibodies, folate, etc.) to target cell receptors overexpressed in cancer cells and the tumor microenvironment [188,215,216]. Cell receptors overexpressed in cancer cells, such as folate-receptor, transferrin-receptor and the EGFR/HER2 receptors are attractive targets because besides the active targeting, their involvement in cell uptake mechanisms may also result in an amplification of the therapeutic effect [217]. However, these cell-receptors are not overexpressed in every cancer type. Another attractive alternative is to target the tumor microenvironment (TME). The TME provides a more general targeting approach since its components/targets are not cancer-type specific. Another advantage is that the need for the particles to diffuse through the tumor can be bypassed by targeting the vasculature directly, and in addition, inhibition of angiogenesis leads to a growth inhibition of the tumor and metastasis [217]. Common overexpressed microenvironment receptors to target are the VEGF, VCAM and αβ-integrins.

Along with the tumor-targeting strategy, physicochemical properties like the ligand density, the size and charge of the NPs, and the physicochemical properties of the targeting ligand, should also be taken into consideration as these parameters will influence the NP stability and targeting properties [188]. In addition, care should also be taken to avoid that high affinity ligands lead to rapid binding of the particles to perivascular cells upon their extravasation to the tumor and limit their in-depth tumor diffusion—a phenomenon nicknamed as the “binding-site barrier” [188].

By using iRGD and cRGD peptides to target integrin receptors (commonly overexpressed in tumor cells [221]) it was demonstrated that RGD-PEG-lipid-DNA NPs accumulated preferentially in tumors compared with normal tissues [171]. However, this enhanced specific targeting of the tumors is also significantly modulated by the remaining physicochemical properties of the particle. Besides the inclusion of the targeting peptides, the particles’ charge, which promotes non-specific interactions with cells, should be minimized by decreasing the charge ratio and liposome charge density. Importantly, it was also found that optimal tumor targeting is achieved for intermediate peptide coverage.

### 8.3. Exploiting Local Stimuli

The tumor and its microenvironment have a series of subtle pathological changes that can be exploited for stimuli-responsive transformations in the particles, leading to improved therapeutic potential. Such special characteristics include a lower pH, higher temperature and overexpression of some proteolytic enzymes [216,217].

We have seen above that pH-responsive cleavable PEG-lipids can be used to enhance the endosomal release of PEGylated lipid-NA nanoparticles. The lower pH of the tumor microenvironment (~6.5–6.8) can also be exploited to cleave PEG from the NPs and facilitate cell uptake. In addition, the particles may contain a cell-penetrating peptide hidden within the PEG coating that becomes exposed when the PEG is removed, improving even further cell uptake [217]. The higher acidity of the tumor microenvironment is also likely to promote the surface charge density of cationic ionizable LNPs, facilitating cell uptake within the tumor.

Other unique pathophysiological conditions of the tumor environment, such as matrix metalloproteinases (MMP) activity, have been explored. MMP activity is tightly regulated in healthy cells; however, expression and activation of MMPs are upregulated in most cancer types owing to their ability to degrade the extracellular matrix, which is involved in angiogenesis, invasion and metastasis [222]. Here, the cleavage of PEG is facilitated upon arrival at the tumor site as a result of the insertion of a MMP-cleavable peptide as a linker between PEG and the nanoparticle [223]. This construct, MMP-cleavable peptide combined with PEG, has been shown to remain efficient in charge shielding and steric stabilization. Upon MMP cleavage, both PEG and the residues are released from the nanoparticle, exposing positive charges which increase the interaction between the nanoparticles and the cellular membrane [223,224,225].

## 9. Prospects

As gene therapy medicines have finally started to be approved for the treatment of patients with monogenic hereditary diseases, a new hope also emerges for cancer treatments. However, given the polygenic nature of cancer, resistance mechanisms and ability to metastasize, a significant amount of progress at the level of our knowledge of cancer, genetics and nucleic acid delivery is still needed until gene therapy can reach a level amenable for routine cancer therapy [5]. Notwithstanding, progress in the field continues accumulating and some clinical trials are starting to show real promise, especially using immunotherapy approaches [7,9,10,11,12,13,14,15].

Given the polygenic nature of cancer, replacing or silencing a single gene may be insufficient and not produce the desirable outcome. In this sense, tumor suppressor genes, such as the *p53* and *mda-7*/IL-24, are able to provide a broader and more versatile means of attacking cancer by inhibiting critical functions to the tumor while being harmless to normal cells [5]. *mda-7*/IL-24 in particular seems especially promising given its potential to treat not only a wide array of solid tumors, but also to treat metastasis, inhibit tumor angiogenesis and stimulate anti-tumor immune response [5]. Other versatile approaches include delivering genes to induce apoptosis or enhance tumor sensitivity to conventional drugs (suicide genes) and silencing oncogenes or genes involved in the cancer pathway with siRNA and miRNAs. The emergence of chemically modified mRNA has also led to improved efficacy and improved safety by eliminating the risk of insertional mutagenesis [12], and the advent of new gene editing systems such as the CRISPR/Cas9 also brings prospects for more permanent oncogene silencing and even gene repairing [42]. These approaches can still suffer from cancer resistance mechanisms, but as our knowledge of cancer increases and new gene targets are identified, new therapeutic NAs can also be synthesized and formulated relatively fast. This will provide relapsing patients with more alternatives and is in contrast with conventional synthetic drugs that take years to develop.

Nevertheless, the high heterogeneity of cancer means that besides the prospect of new therapeutic NAs, the carrier also needs to be flexible. Non-viral lipid-based systems, despite their still relatively low in vivo transfection efficiency, have been making significant improvements in the last decades. Importantly, their versatility, affordability, the potential for scaling up, and modular approach design that allows to rapidly tune the structure, physical properties and functionalization, are poised to go hand-in-hand with advances in our knowledge of cancer and genetics. As these lipid nanocarriers continue evolving, the prospect is that they shall be able to deliver NAs in formulations optimized for specific tumors and specific patients.

This seemingly personalized cancer treatment is in fact already materializing as companies, like Moderna and BioNTech, are developing cancer vaccines personalized for each patient. Early-stage clinical trials are showing promising results [9,12]. The strategy consists in elucidating for each patient the tumor mutations producing antigens that are most likely to stimulate the immune system to fight cancer. mRNA encoding these antigens is synthesized and administered to the patient to stimulate the immune system against cancer and the whole process from tumor sequencing to delivery of the mRNA vaccine can be optimized to 6–7 weeks [12]. Importantly, as T cells of the effector memory type seem to be induced, this approach should be capable of controlling the outgrowth of micrometastases, and as the tumor changes with time, the personalized patient vaccine composition can also be adjusted [13]. Because of the role of T cells in cancer vaccines, combinatorial strategies with immune checkpoint inhibitors are also a promising approach [12,13]. Additionally, customizable lipid-NA nanoparticles could prove important to target different immune cells or organs (e.g., spleen, lymph nodes and bone marrow). Importantly, one of the recent exciting results in in vivo mice studies and early-stage clinical trials has found that dendritic cells can be targeted precisely and efficiently after intravenous administration of negatively charged mRNA lipoplexes without the need for functionalization with PEG or targeting ligands [8,15]. This highlights that knowledge about the interactions of lipid systems with biologic systems in vivo is still incomplete and further progress is desirable to fully harness their potential.

In the quest to develop more potent formulations to treat cancer, high-throughput particle assembly methods have been utilized to identify novel and more potent ionizable lipids for gene therapy [185]. For now, most of these searches have focused on changing the ionizable lipid while keeping the remaining LNP composition in terms of cholesterol, DSPC and PEG-lipid fixed. Older work has shown that the transfection of lipid-based particles can be significantly improved by manipulation of the lipoplex membrane charge density [130,193] (Figure 7), and more recent work on mRNA ionizable LNPs [124] seems to indicate the same trend as the area per DSPC was identified as a critical parameter. High-throughput screening approaches that also try to identify the influence of various compositional parameters (e.g., charge ratio and membrane charge density) on the transfection efficiency in ever more realistic in vitro models (including the influence of serum and protein absorption) may reveal unexpected behavior to be explored for improved therapeutic outcomes. This task may be facilitated by new in vitro tumor-on-a-chip platforms that allow mimicking several important aspects of the tumor microenvironment, and can be used for high-throughput screening of new anticancer formulations [226,227]. Simultaneously, new microfluidic methods are also now in use that allow the preparation of lipid-NA NPs in a more rapid, controlled and reproducible away, promising to optimize the assembly of these particles even further and accelerate the discovery of more potent formulations [175,180,183,184,185,228,229,230].

While the path to obtaining efficient gene therapeutic approaches to treat cancer is still long and tenuous, encouraging results in clinical trials, especially in personalized cancer vaccines, start to finally show [8,9,12]. As our knowledge and progress in lipid-NA delivery technology continues evolving [19,25,231], the prospect of potentiating these encouraging early-stage clinical results sets a new hope for the future.

## 10. Conclusions

In this review, we provided a simplified fundamental overview on the formation of lipid-NA complexes and how their structures and biophysical properties can be tuned to enhance gene delivery. We described fundamental concepts such as the universal transfection curve in simplified in vitro studies and how intracellular and tumor properties and stimuli can be exploited to improve delivery efficiency. We also briefly described some cancer therapeutic strategies involving the delivery of DNA and mRNA to supplement tumor suppressor genes and RNA interference of cancer-critical genes, both of which have a broad activity in the cancer pathways. New advances in cancer immunotherapy, especially in the use of personalized cancer vaccines using mRNA delivery, is also exciting. The prospect of using the patients’ own immune system to fight cancer in a delocalized way, keeping the resurgence of metastases under control, makes immunotherapy one of the most promising approaches to treat cancer. As our knowledge and progress in lipid-NA delivery technology continues evolving, the prospect of potentiating these exciting therapeutic strategies sets a new hope for the future.
